# Linked genetic loci and genotype-dependent temperature effects shape craniofacial morphology in medaka

**DOI:** 10.1093/g3journal/jkag094

**Published:** 2026-04-09

**Authors:** Minori Shinya, Tetsuaki Kimura, Takafumi Ikeda, Ryohei Nakamura, Hiroyuki Takeda, Kiyoshi Naruse

**Affiliations:** Department of Biology, Keio University, Yokohama, Kanagawa 223-8521, Japan; Medical Genome Center, Research Institute, National Center for Geriatrics and Gerontology, Obu, Aichi 474-8511, Japan; Faculty of Life Sciences, Kyoto Sangyo University, Kyoto 603-8555, Japan; Institute for Protein Dynamics, Kyoto Sangyo University, Kyoto 603-8555, Japan; Department of Biological Sciences, Graduate School of Science, The University of Tokyo, Tokyo 113-0033, Japan; Faculty of Life Sciences, Kyoto Sangyo University, Kyoto 603-8555, Japan; Laboratory of Bioresources, National Institute for Basic Biology, Okazaki, Aichi 444-8585, Japan

**Keywords:** craniofacial morphology, complex traits, genetic factors, rearing temperature, *Oryzias*

## Abstract

Craniofacial morphology is a fundamental and complex trait, vital for survival and crucial for social interactions such as individual recognition and mate selection. While its overall form is conserved within a species, significant individual diversity exists, controlled by both genetic and environmental factors. To elucidate its genetic basis, we employed quantitative trait locus (QTL) analysis using F_2_ progeny obtained from 2 medaka inbred strains (HNI-II and HdrR-II1), identifying multiple genetic loci that significantly contribute to variation in craniofacial morphological traits. Further fine-mapping using congenic strains for 2 selected traits demonstrated that each phenotype is influenced by multiple QTLs within a single chromosome. Specifically, a cluster of 3 candidate QTLs on chromosome 6 is found to regulate the head length-to-height ratio (L33), potentially involving interactions to affect the phenotype. High-resolution Hi-C analysis revealed physical chromatin interactions among these clustered QTLs, suggesting that spatial genomic architecture facilitates their functional interactions. Our study also revealed that L33 is sensitive to rearing temperature. We observed a genotype-dependent response, in which low temperature (23 °C) significantly altered phenotypes in HdrR-II1 but not in HNI-II. Our findings suggest that craniofacial diversity is shaped by the complex interplay of multiple QTLs and temperature effects, highlighting medaka as a powerful model for investigating how genetic interactions and environmental plasticity shape morphological diversity.

## Introduction

The craniofacial structure is a critical part of body containing many vital structures such as the brain, eyes, mouth, and nose. Abnormalities in craniofacial morphology sometimes make it impossible for the affected individuals to survive (Roberts 1967; Chiang et al. 1996; Neuhauss et al. 1996). While the overall morphology is species-specific, individual variation also exists. Humans use differences in facial shape as a means of individual recognition, and female medaka fish have been shown to choose familiar male fish for mating by visual information around the head region (Wang and Takeuchi 2017). Thus, craniofacial morphology is an important and intriguing trait that influences both individual survival and social interactions.

Individual differences in craniofacial morphology are thought to be influenced by both environmental and genetic factors. Analyses to identify gene(s) contributing to the morphological variations have been conducted extensively in model animals such as mice and fish (Klingenberg et al. 2001, 2004; Dohmoto et al. 2002; Kimura et al. 2007), as well as in humans (Liu et al. 2012; Paternoster et al. 2012; Adhikari et al. 2016; Cole et al. 2016; Shaffer et al. 2016; Lee et al. 2017; Cha et al. 2018; Claes et al. 2018; Qiao et al. 2018; Wu et al. 2019; Kim et al. 2024). Genomic regions and/or candidate genes associated with craniofacial morphology have been reported, and several genes such as *DCHS2*, *PAX3*, and *SOX9* have been replicated in the previous studies. These candidate genes include various types of genes, such as transcription factors and growth factors, a part of which have been shown to function in craniofacial development, indicating that numerous genetic factors are involved in the formation of individual differences in craniofacial morphology.

Medaka (*Oryzias latipes* and *Oryzias sakaizumii*) is a small fish widely distributed in temperate regions of Japan (Iwamatsu 1997) and, owing to its high fertility, ease of breeding, and the availability of numerous inbred strains, has been extensively used for statistical genetic analyses of quantitative traits (Kimura et al. 2012; Tsuboko et al. 2014). We previously identified significant differences in craniofacial morphology between northern and southern inbred strains and, for the first time, applied quantitative trait locus (QTL) analysis to this medaka system (Kimura et al. 2007). In this study, we analyzed 89 craniofacial traits by interval mapping in 184 F_2_ individuals derived from the 2 inbred strains, HNI-II and HdrR-II1, and mapped 66 of these traits to specific chromosomal regions.

In this study, we examined craniofacial traits from our previous analysis that showed no sexual dimorphism and displayed pronounced differences between the 2 inbred strains. Through refined interval mapping with an increased F_2_ sample size and subsequent phenotypic analysis using congenic strains, we successfully mapped 2 selected traits (L33 and D23) and found that, for each trait, multiple QTLs are located on a single chromosome. For L33 (the head length-to-height ratio), Hi-C analysis suggested functional interactions among the candidate QTLs. Additionally, we found that rearing temperature influenced L33 in a genotype-dependent manner.

## Materials and methods

### Strains

Two medaka inbred strains, HNI-II (Strain ID: IB176) and HdrR-II1 (Strain ID: IB178) strains (Hyodo-Taguchi 1996) were supplied by NBRP Medaka (https://shigen.nig.ac.jp/medaka/), and used in this study. HNI-II had been established from a northern Japanese population (*O. sakaizumii*), and HdrR-II1 was from a southern Japanese population (*O. latipes*). Two pairs of HNI-II males and HdrR-II1 females, and 3 pairs of HdrR-II1 males and HNI-II females were crossed to generate F_1_ progeny. By intercrossing 5 pairs of the F_1_ fish, 184 F_2_ progeny were obtained. Those F_2_ progeny were used for the interval mapping together with another 184 F_2_ progeny analyzed in the previous study (Kimura et al. 2007). Two consomic and 17 congenic strains substituted a part of genome from the HdrR-II1 into the HNI-II origin was used for phenotypic analysis. Congenic strains used for narrowing QTLs are referred to by abbreviations throughout this study, with their full names provided in Supplementary Tables 1 and 2. HdrR-II1-Chr6^HNI-II and HdrR-II1-Chr22^HNI-II strains had been established as consomic strains substituted the entire chromosome (Chr) 6 and 22, respectively (Shinya et al. 2023). HdrR-II1-Chr5A^HNI-II, C6A, C15A, and C15B strains were generated using the high-speed congenic system (Shinya et al. 2023) with some additional crossing. In HdrR-II1-Chr5A^HNI-II, the genomic region between MM03B11K and MM04A06K was HNI-II-homozygous and the substituted regions in the rest 3 strains were shown in Supplementary Tables 1 and 2. C6C, C6E, and C6F strains (Supplementary Table 1) were prepared by back-crossing HdrR-II1-Chr6^HNI-II into HdrR-II1 for 2 or more generations, and the offspring that carried a desirable recombination were finally intercrossed to make homozygotes for the substituted segment. The rest of congenic strains with a part of Chr 6 substitution (5 strains: Supplementary Table 1) were generated by back-crossing C6C medaka into HdrR-II1, following the same steps as described in the above. Five more congenic strains with a part of Chr 15 substitution (Supplementary Table 2) were generated by back-crossing C15A medaka into HdrR-II1, following the same steps as described in the above. All experimental procedures were approved by the Keio University Institutional Animal Care and Use Committee (No. 14033, 17009, A2022-092) and carried out according to the Institutional Guidelines on Animal Experimentation at Keio University.

### Breeding and phenotypic analysis

Fish used for genetic studies were bred in an in-house facility at 27.5 °C in a constant recirculating system on a 14 h light/10 h dark. The inbred medaka used in the experiment to study the effects of rearing temperature were bred in the above condition until 2 months post fertilization (mpf), and then, placed in a tank without water circulation at 3 temperatures (23 °C, 27.5 °C, and 32 °C). Craniofacial morphology of adult fish at 4 mpf was quantified basically using the method described in a previous study (Kimura et al. 2007). Phenotypic comparisons of 2 strains or groups employed Welch's *t*-test. For the multiple comparisons, significant *P*-values were adjusted using Bonferroni's correction.

### Primer design

We designed 7 RFLP (restriction fragment length polymorphism) and 12 PLP (PCR product length polymorphism) markers (Supplementary Table 3). We first compared genome sequences of the targeted region between the HdrR-II1 and HNI-II in order to identify polymorphisms, using UT Genomebrowser (not currently available) or BLASTn on Ensembl (http://asia.ensembl.org/index.html). To amplify the region containing the polymorphism, forward and reverse primers were designed using software or websites such as Genetyx software (Genetyx, Tokyo, Japan) and Primer3web (https://primer3.ut.ee/). Then, both forward and reverse primers were confirmed not to map to any other region of the medaka genome using BLASTn on Ensembl, or the designed primer pairs were verified to amplify a single copy on the medaka genome using in silico PCR on UCSC Genome Browser (https://genome.ucsc.edu/cgi-bin/hgPcr). Each primer pair was experimentally tested with HNI-II fish, HdrR-II1 fish, and F_1_ progeny to determine whether it would work as a codominant genetic marker.

### DNA extraction and genotyping

The genomic DNA of F_2_ progeny were extracted as described in the previous study (Kimura et al. 2005). For the selection of mating fish to establish congenic strains, the genomic DNA of backcross or intercross progeny were prepared from fin-clips by the method shown in Shinya et al. (2023). The genotype of F_2_ progeny was determined as described previously (Kimura et al. 2004, 2005; Naruse et al. 2004). Genotyping a RFLP marker, MF01SSA033C03, for congenic strains was performed by the method described in Naruse et al. (2004), and DNA fragments were detected by QIAxcel system (QIAGEN N.V., Venlo, Netherlands). PLP markers for congenic strains were basically genotyped as described in Kimura et al. (2005): AmpliTaq Gold DNA polymerase (Applied Biosystems, Foster City, CA) or ExTaq polymerase (TaKaRa, Kusatsu, Japan) were used for PCR reactions, and the PCR fragments were detected by ABI Prism 3730 DNA analyzers (Applied Biosystems, Foster City, CA) or QIAxcel system.

### QTL mapping

A total of 277 genetic markers, composed of 109 SNPs (Kasahara et al. 2007), 135 PLP markers (Kimura et al. 2004, 2005; Naruse et al. 2004), and 33 RFLP markers (26 markers from Naruse et al. (2004) and 7 markers designed in this study), were used for QTL mapping of the craniofacial morphology. The genotype data of SNPs were from Kasahara et al. (2007). Those of the F_2_ progeny for the PLP markers examined by Kimura et al. (2005) were used in this study as is, and the genotype data for the newly added F_2_ progeny and PLP makers were obtained through the method described in the above. All RFLP markers were genotyped in this study. Genotype data were shown in Supplementary Table 4. Based on the genotype data, a linkage map was constructed using MAPMAKER/EXP 3.0b (Lander et al. 1987). Recombination frequencies were converted to map distance (cM) by using Haldane's map function. The linkage map covered 1467.4 cM of the medaka genome with an average interval distance of 5.80 ± 6.68 cM. Craniofacial traits were mapped on the linkage map by interval mapping using R/qtl (Broman et al. 2003). The significance threshold of a logarithm of odds (LOD) score was 2.8, which indicates the chance of a false positive occurring anywhere in the medaka genome at most 5% (Lander and Botstein 1989). Composite interval mapping which attempts to separate and isolate individual QTL effects (Zeng 1993, 1994), was performed using the Windows program QTL Cartographer 2.5 (Silva Lda et al. 2012).

### Comparative genome analysis

For the comparative genome analysis, we used the recently established telomere-to-telomere (T2T) quality genome assemblies of the medaka HdrR-II1 (Olatipes_Hd-rR_3.1) and HNI-II (Olatipes_HNI_3.1) strains (Suzuki et al. 2025). Gene names and GO terms were annotated using eggNOG-mapper v2 (Cantalapiedra et al. 2021), and comprehensive gene lists were constructed for the targeted genomic regions. To perform synteny analysis, the genomic sequences surrounding the target regions of the HdrR-II1 and HNI-II strains were aligned using minimap2 (v2.30) (Li 2018). Syntenic regions and structural rearrangements were identified with SyRI (v1.7.1) (Goel et al. 2019), and the results were visualized using plotsr (v1.1.1) (Goel and Schneeberger 2022). In addition, VISTA plots (Frazer et al. 2004) were generated to detect small insertions and deletions that may not have been captured by SyRI.

### Hi-C analysis

For HdrR-II1 embryonic fibroblasts, a previously published Hi-C contact map (Nakamura et al. 2021 , DDBJ BioProject Accession: PRJDB7492), generated using the ASM223467v1 medaka reference genome, was used. In that study, fibroblast cells were cultured at 30 °C. Hi-C contact maps of 6-somite stage embryos from HdrR-II1 and HNI-II strains (DDBJ BioProject Accession: PRJDB19938), previously generated and described in Suzuki et al. (2025) were also used. In that study, embryos were staged based on the number of somites, and rearing temperature was not strictly controlled. These embryo Hi-C maps were generated using the T2T genome assemblies, Olatipes_Hd-rR_3.1 and Olatipes_HNI_3.1 for HdrR-II1 and HNI-II, respectively (Suzuki et al. 2025). Topologically associating domains (TADs) shown in Fig. 4 were manually annotated in this study by visual inspection of the fibroblast Hi-C contact map.

## Results

### QTL mapping analysis

Interval mapping using 368 F_2_ progeny derived from HNI-II and HdrR-II1 strains was performed to clarify the genomic regions involved in the craniofacial traits. The traits analyzed in this study were selected from 125 traits described in Kimura et al. (2007) that showed no sexual dimorphism but showed differences between the inbred strains. Those were 107 traits (33 lateral view traits, 51 dorsal view traits, and 23 ventral view traits, Supplementary Table 5) that followed a normal distribution in the F_2_ progeny; 97 out of 107 traits were mapped on the medaka genome, and a total of 293 significant QTLs with LOD score >2.8 were found (Supplementary Table 6). For the top 20 QTLs with high LOD scores, we showed the percentage variance explained by the QTL and the effect of the allele at the marker nearest the estimated QTL (Table 1). The highest LOD score was obtained in a ventral view trait V13, which was mapped to Chr 5 with a LOD score of 7.87. There was an additional 3 QTLs mapped with a LOD score of 7.0 or higher.

**Table 1.** QTLs mapped with top 20 LOD scores.

						N/N^a^		N/R^b^	R/R^c^		*P* ^d^
Trait	Chr	Position (cM)	LOD	% Var^e^	Nearest marker	Mean ± SE		Mean ± SE	Mean ± SE		
V13	5	25.2	7.87	10.3	OLa3008b	1.421 ± 0.017	*	1.353 ±0.011	1.290 ± 0.015	*	0.000000042
V12	5	24.8	7.50	9.8	OLa3008b	1.133 ± 0.011	*	1.087 ± 0.007	1.048 ± 0.011	*	0.00000011
L33	6	33.8	7.27	9.2	Scaffold11I	1.861 ± 0.009		1.888 ± 0.006	1.931 ± 0.008	*	0.00000010
D23	15	41.4	7.05	10	MF01SSA028G08	1.189 ± 0.006		1.173 ± 0.003	1.145 ± 0.006	*	0.00000014
D24	15	46.2	6.91	8.6	MF01SSA028G08	1.058 ± 0.006		1.041 ± 0.004	1.010 ± 0.006	*	0.00000014
L29	6	34	6.79	8.5	MF01SSA031E04	1.765 ± 0.009		1.778 ± 0.006	1.827 ± 0.008	*	0.00000024
V18	23	19	6.75	10.2	TUN9_030	1.175 ± 0.004	*	1.158 ± 0.003	1.149 ± 0.003		0.0000013
V20	13	19.8	6.64	9.2	MM05D11K	1.037 ± 0.002		1.031 ± 0.001	1.023 ± 0.002	*	0.0000010
D28	15	43.2	6.53	9	MF01SSA028G08	3.198 ± 0.034		3.273 ± 0.023	3.484 ± 0.048	*	0.00000037
D29	22	27.4	6.51	7.9	MF01SSA010H01	1.943 ± 0.007	*	1.989 ± 0.006	2.007 ± 0.009		0.00000055
V17	17	15.1	6.45	7.8	Scaffold332	1.137 ± 0.003	*	1.123 ± 0.002	1.112 ± 0.004	*	0.00000040
D31	15	29.8	6.45	10.2	MM02D04K	1.344 ± 0.009	*	1.311 ± 0.005	1.287 ± 0.007	*	0.0000020
D6	15	46.4	6.44	7.8	MF01SSA028G08	1.490 ± 0.009		1.476 ± 0.005	1.432 ± 0.008	*	0.00000041
D39	12	43.4	6.32	10.5	MM05B02K	13.02 ± 0.24		12.37 ± 0.17	11.29 ± 0.26	*	0.0000057
D41	12	43.4	6.32	10.5	MM05B02K	12.02 ± 0.24		11.37 ± 0.17	10.29 ± 0.26	*	0.0000057
D43	12	43.4	6.24	10.3	MM05B02K	10.49 ± 0.20		9.96 ± 0.14	9.08 ± 0.21	*	0.0000060
L29	4	27.8	6.17	7.9	MM01F02K	1.818 ± 0.009		1.792 ± 0.006	1.753 ± 0.008	*	0.00000094
V5	5	25.6	6.10	7.8	OLa3008b	1.561 ± 0.014	*	1.607 ± 0.009	1.674 ± 0.019	*	0.0000014
D29	15	8.6	5.88	8.7	TUN10_099	2.013 ± 0.008	*	1.982 ± 0.006	1.953 ± 0.007	*	0.0000093
V17	17	37.1	5.78	7	MF01SSA033A11	1.137 ± 0.003	*	1.123 ± 0.008	1.114 ± 0.004		0.0000019

^a^Phenotypic value of the homozygotes for the HNI-II allele at the nearest marker. All were significantly different from R/R mean (Welch's *t*-test, Bonferroni adjusted *P* < 0.0167). Asterisks indicate significant difference from N/R mean (Welch's *t*-test, Bonferroni adjusted *P* < 0.0167).

^b^Phenotypic value of the heterozygotes at the nearest marker.

^c^Phenotypic value of the homozygotes for the HdrR-II1 allele at the nearest marker. Asterisks indicate significant difference from N/R mean (Welch's *t*-test, Bonferroni adjusted *P* < 0.0167).

^d^ *P*-value of one-way analysis of variance.

^e^Percentage of the phenotypic variance that can be explained by the QTL.

### Validation of QTLs

We selected a subset of the identified QTLs for validation. This included 4 QTLs with LOD scores greater than 7.0 (V13 on Chr 5, V12 on Chr 5, L33 on Chr 6, and D23 on Chr 15), as well as 2 QTLs identified for D29 (on Chr 15 and 22). We chose the 2 D29 QTLs because they were mapped to only 2 loci by interval mapping, and both showed high LOD scores that placed them within the top 20 (Table 1).

To validate the identified QTLs, we compared specific congenic strains (Supplementary Fig. 1) with the HdrR-II1 strain. For V13 (Chr 5), L33 (Chr 6), and D23 (Chr 15), significant phenotypic differences were observed between the respective congenic and HdrR-II1 strains (Supplementary Table 7). These phenotypic shifts were consistent with the direction of the HNI-II allele effects estimated in Table 1. Regarding D29 (Chr 15), a significant difference was confirmed in the C15A strain but not in C15B (Supplementary Table 7). However, when comparing the parental strains (HNI-II and HdrR-II1) in our current samples, results for L33 and D23 were consistent with previous findings (HNI-II > HdrR-II1; Kimura et al. 2007), whereas the phenotypic relationship was reversed for V13, and no significant difference was detected for D29 (Supplementary Table 7). Consequently, we considered V13 and D29 to be unstable, likely fluctuating due to subtle differences in rearing conditions, and thus precluded them from further analysis. Meanwhile, no associations were confirmed for V12 on Chr 5 or D29 on Chr 22, as their respective congenic strains showed no significant differences. Therefore, we proceeded with further analysis focusing on L33 (Chr 6) and D23 (Chr 15).

### Narrowing the QTL for L33 on Chr 6

**Fig. 1.**
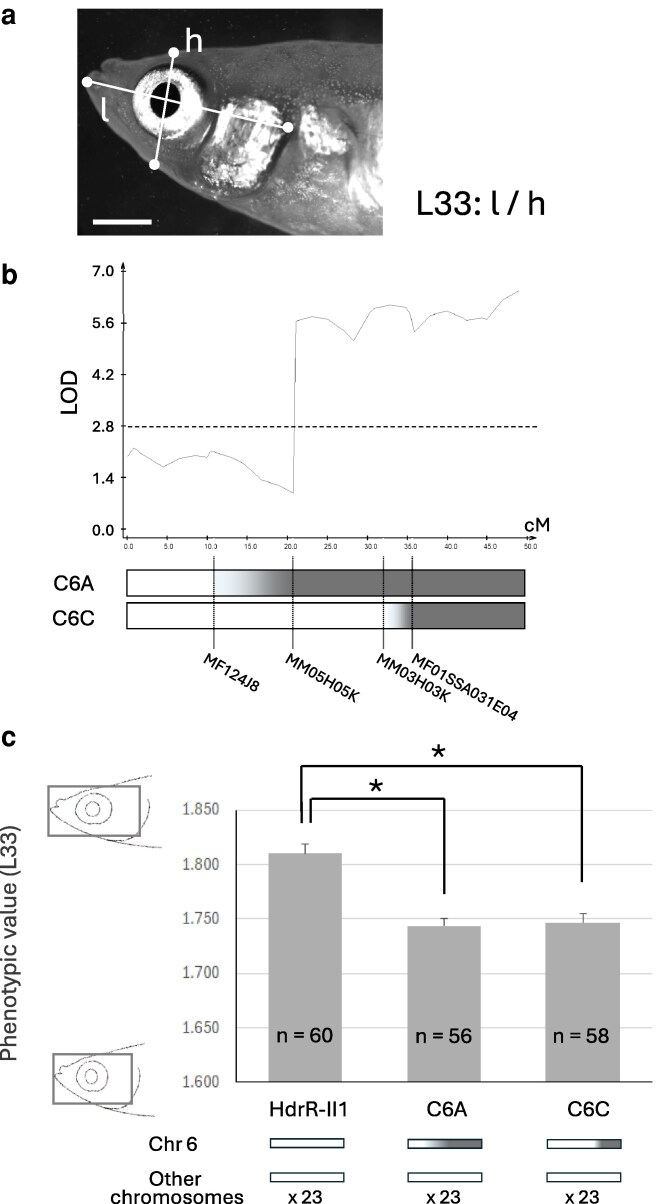
Narrowing of the QTL for a lateral view trait L33 on Chr 6. a) Trait L33. The trait was calculated by head length (l) divided by head height (h). Bar, 3 mm. b) Position of the QTL found by composite interval mapping, and diagram of congenic strains used for narrowing. The dashed line indicates the significance of LOD threshold. A part of Chr 6 of HdrR-II1 (shown in white) was substituted with DNA from HNI-II (shown in gray) in each strain. c) Phenotypic values of L33 in the 3 strains. Asterisks indicate significant differences (Welch's *t*-test, Bonferroni adjusted *P* < 0.025). Error bars, S. E.

A lateral view trait, L33, represents the ratio of head length to head height (Fig. 1a). Composite interval mapping restricted the association of trait L33 to approximately half of Chr 6 (Fig. 1b). Two congenic strains for this area (C6A and C6C) exhibited significantly smaller phenotypic values than the HdrR-II1 (Fig. 1c). Because all other chromosomes and regions are derived from HdrR-II1, this result localized the QTL to a region between marker MM03H03K and the chromosome terminus, spanning ∼6 Mbp.

**Fig. 2.**
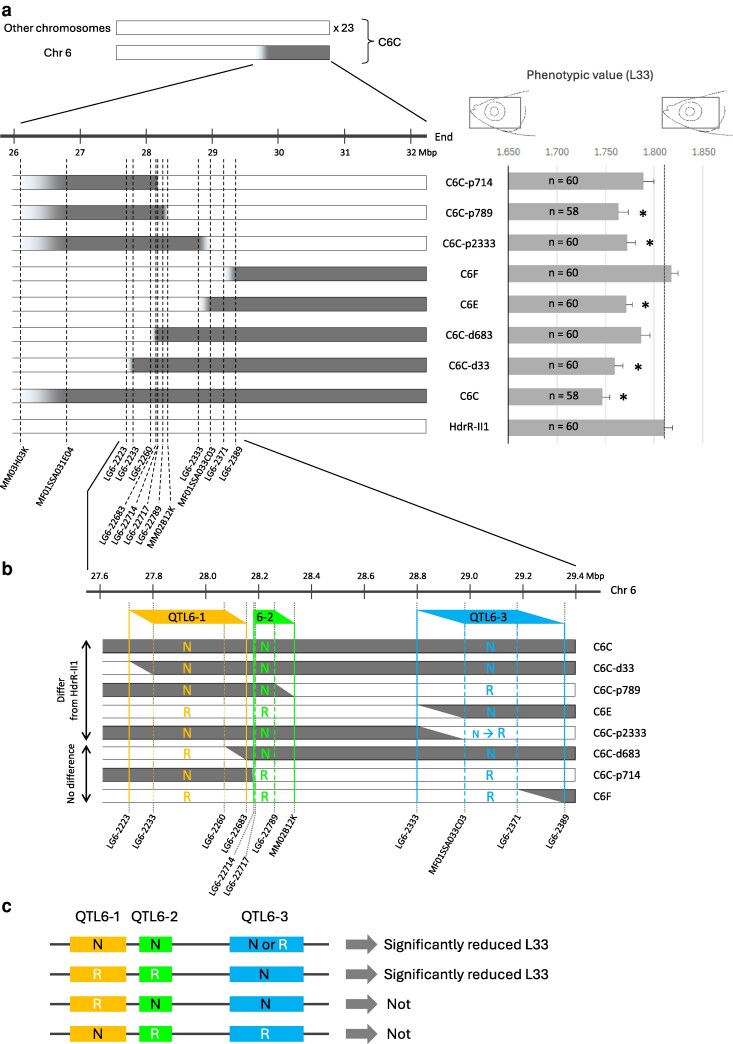
Phenotypic analysis of congenic strains to narrow the QTL for L33 within the region covered by HNI-II-derived genome in the C6C strain. a) Diagram of congenic strains used for narrowing (left) and phenotypic values (right) for L33. Physical position was derived from the HdrR-II1 genome (ASM223467v1). Chromosomal regions that were substituted with DNA from HNI-II are shown in gray, and regions in white originated from HdrR-II1. Asterisks indicate significant differences when compared with HdrR-II1 (Welch's *t*-test, Bonferroni adjusted *P* < 0.0063). Error bars, S. E. b) The region between markers LG6-2223 and LG6-2389 to show 3 candidate QTLs (QTL6-1, QTL6-2, and QTL6-3). The phenotypic loss observed between specific strains defined those as follows. QTL6-1: the region from the HNI-II/HdrR-II1 boundary (N/R boundary) of C6C-d33 to the N/R boundary of C6C-d683. QTL6-2: the region from the N/R boundary of C6C-p714 to the N/R boundary of C6C-p789. QTL6-3: the region from the N/R boundary of C6E to the N/R boundary of C6F. “N” represents HNI-II derived genome, and “R” represents HdrR-II1 derived genome in each QTL. c) Summary of the effect of the 3 candidate QTLs.

Subsequent fine-mapping using 7 congenic strains revealed that 4 strains (C6C-p789, C6C-p2333, C6E, and C6C-d33) exhibited significantly reduced L33 values, whereas the remaining 3 (C6C-p714, C6F, and C6C-d638) did not (Fig. 2a). If a single locus within this 6 Mbp region were responsible for the L33 variation, a common genomic interval should be shared by all 4 affected strains. However, no such common interval was identified, indicating that the reduced phenotype in C6C is not driven by a single, independently acting locus. Furthermore, because the phenotypic value of C6F was nearly equivalent to that of HdrR-II1 (Fig. 2a), Genetic loci influencing L33 are unlikely to reside in the region downstream of marker LG6-2389 on Chr 6. Taken together, these results suggest that multiple QTLs are present within the chromosomal segment between MM03H03K and LG6-2389 on Chr 6 (∼3.5 Mbp).

### Three candidate QTLs for L33 on Chr 6

Detailed comparison of the HNI-II-derived genomic regions between strains with and without significant differences from HdrR-II1 noticed 3 distinct loci possibly influencing the L33 phenotype (Fig. 2b). The distinct phenotypic loss observed between specific strain pairs defined these regions: (i) QTL6-1 (<444 kbp, markers LG6-2223–LG6-22683): defined by the difference between C6C-d33 and C6C-d683. (ii) QTL6-2 (<152 kbp, markers LG6-22714–MM02B12K): defined by C6C-p789 vs C6C-p714. (iii) QTL6-3 (<561 kbp, markers LG6-2333–LG6-2389): defined by C6E vs C6F.

Notably, some strains in which only some of these loci were originated from HNI-II—such as C6C-p714 (HNI-II at QTL6-1 only) and C6C-d683 (HNI-II at both QTL6-2 and QTL6-3 but not at QTL6-1)—showed no significant difference from HdrR-II1 (Fig. 2b). This suggests that these loci function in combination, and no single locus is sufficient to produce the phenotype. The effects of these loci appear to follow 2 rules: First, QTL6-1 and QTL6-2 significantly affect L33 when both carry HNI-II alleles; all strains with both loci originated from HNI-II exhibited significantly reduced phenotypic values, regardless of the QTL6-3 origin (Fig. 2c). Second, the effect of QTL6-3 appears to be conditional on the genotype of the other 2 loci. Specifically, the HNI-II-derived QTL6-3 significantly reduced the phenotype only when origins of QTL6-1 and QTL6-2 were concordant (Fig. 2c) (ie both derived from HdrR-II1, as in C6E, or both from HNI-II, as in C6C-d33; Fig. 2b). In contrast, no significant reduction occurred in strains such as C6C-d683, where the genomic origins of QTL6-1 and QTL6-2 were discordant (Fig. 2b).

### Genes in the 3 candidate QTLs for L33

Since the 3 QTLs were fine-mapped to narrow intervals (<600 kbp), we examined the gene content within each region. Based on annotation of the medaka T2T genome sequence (see Materials and methods), QTL6-1 and QTL6-3 each harbored around 30 genes, whereas QTL6-2 contained only several genes, including *fibroblast growth factor 6* (*fgf6*) (Supplementary Table 8). Comparison between the HdrR-II1 and HNI-II genomes revealed amino acid substitutions in most of these genes (Supplementary Table 8). Although no large-scale genomic structural differences were observed in the vicinity of these QTLs (Supplementary Fig. 2), numerous sequence discrepancies were detected within each QTL region between the 2 inbred genomes (Supplementary Figs. 3, 4). Interestingly, while *fgf6* carried no nonsynonymous substitutions (Supplementary Table 8), we identified a ∼2 kbp sequence insertion located ∼2 kbp upstream of the gene in the HdrR-II1 genome, as well as a ∼1.6 kbp insertion within the second intron in the HNI-II genome (Supplementary Fig. 5).

### Narrowing the QTL for D23 on Chr 15

**Fig. 3.**
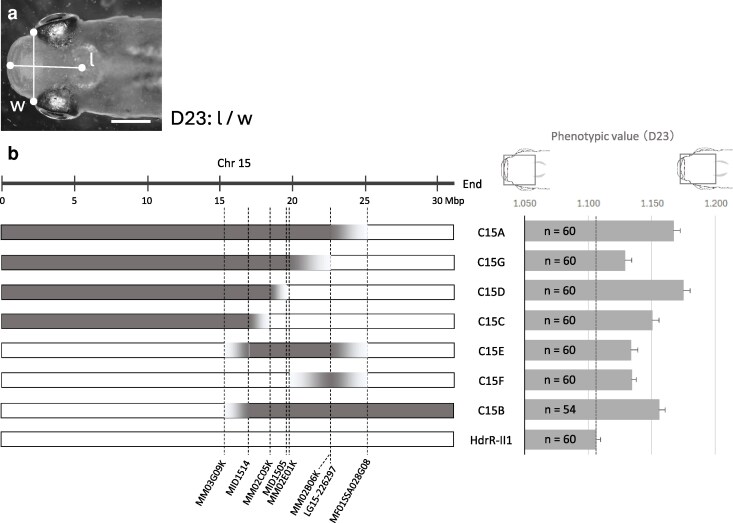
Narrowing of the QTL for a dorsal view trait D23 on chr 15. a) Trait D23. The trait was calculated by the anterior head length (*l*) divided by the head width at the anterior orbital level (*w*). Bar, 3 mm. b) Diagram of congenic strains used for narrowing (left) and phenotypic values for D23 (right). Physical position was derived from the HdrR-II1 genome (ASM223467v1). Chromosomal regions that were substituted with DNA from HNI-II are shown in gray, and regions in white originated from HdrR-II1. All congenic strains were significantly different from the HdrR-II1 (Welch's *t*-test, Bonferroni adjusted *P* < 0.0071). Error bars, S. E.

In parallel with the analysis of L33, we performed fine-mapping of the QTL for D23 on Chr 15. D23 is a dorsal view trait representing the ratio of the anterior head length to the head width at the anterior orbital level (Fig. 3a). In the validation study, both C15A and C15B strains exhibited significantly larger phenotypic values than HdrR-II1 (Supplementary Table 7), thereby narrowing the candidate region to the shared interval between markers MM03G09K (15.28 Mbp) and MF01SSA028G08 (25.14 Mbp) (Supplementary Fig. 1d). We established 5 congenic strains with HNI-II/HdrR-II1 genomic boundaries within this region and compared their phenotypes to that of the HdrR-II1 strain. As shown in Fig. 3b, all the strains examined showed larger phenotypic values than the HdrR-II1 strain, and the differences were statistically significant (Welch's *t*-test, Bonferroni adjusted *P* < 0.0071).

Given that there was no single genomic region commonly replaced by the HNI-II-derived genome in all of the examined congenic strains, it became clear that multiple QTLs on Chr 15 contribute to the phenotype. One QTL is likely located between marker MM02E01K (19.73 Mbp) and MF01SSA028G08 (25.14 Mbp), reflecting the region replaced with the HNI-II-derived genome in the C15F strain. Furthermore, since the C15C strain showed a significant difference from the HdrR-II1 strain even though the region above (between marker MM02E01K and MF01SSA028G08) was not HNI-II-derived, it suggests that another QTL exists in a region, starting from marker MM02C05K (18.46 Mbp) and extending upstream. For practical reasons, we did not pursue narrowing these QTLs to the same level of detail as L33.

### Chromatin contacts around the 3 candidate QTLs

The fine-mapping results for L33 described above suggested interactions among the 3 QTLs, QTL6-1, QTL6-2, and QTL6-3. These were further examined using Hi-C analysis. We re-analyzed previously published Hi-C datasets from medaka HdrR-II1 fibroblasts (Nakamura et al. 2021) and embryos of the HdrR-II1 and HNI-II strains (Suzuki et al. 2025). We primarily focused on high-resolution Hi-C data from HdrR-II1 embryonic fibroblasts, as this data have the highest resolution, and, where appropriate, referred to data from 6-somite stage embryos from HdrR-II1 and HNI-II strain.

**Fig. 4.**
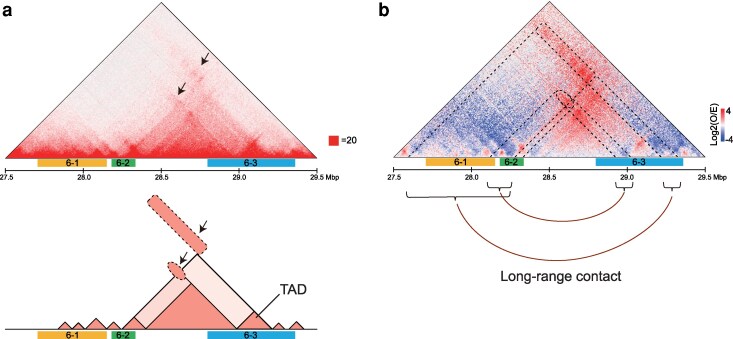
Potential physical interactions of the 3 candidate QTLs (QTL6-1, QTL6-2, and QTL6-3) for L33. Chromatin contacts were examined by high-resolution Hi-C analysis of HdrR-II1 embryonic fibroblasts at 5-kb resolution. a) Heat map of normalized Hi-C contacts (top) and schematic representation of the contacts (bottom). In addition to several TADs, 2 types of long-range contacts were observed (arrows). b) Observed/expected (O/E) contact map. Physical position was derived from the HdrR-II1 genome (ASM223467v1).

Our analysis revealed that the interval spanning from the latter half of QTL6-2 to the initial two-thirds of QTL6-3 resides within a single TAD; defined as genomic intervals with enhanced internal contact frequencies (Fig. 4a). Notably, the observed/expected (O/E) contact map (Fig. 4b) highlighted 2 types of long-range contacts extending from the QTL6-3 region toward broader regions that include QTL6-1 and QTL6-2 (Fig. 4b). These contact patterns were consistently observed not only in fibroblasts but also in medaka embryos (stage 21, 6-somite stage) of both the HdrR-II1 and HNI-II strains albeit at lower resolution (Supplementary Fig. 6). In general, the majority of TADs are known to be shared among cell types (Rao et al. 2014; Bonev and Cavalli 2016). Together, these chromatin contact patterns are consistent with the genetic evidence presented above and support cooperative interactions among the 3 QTLs.

### Effect of rearing temperature

**Fig. 5.**
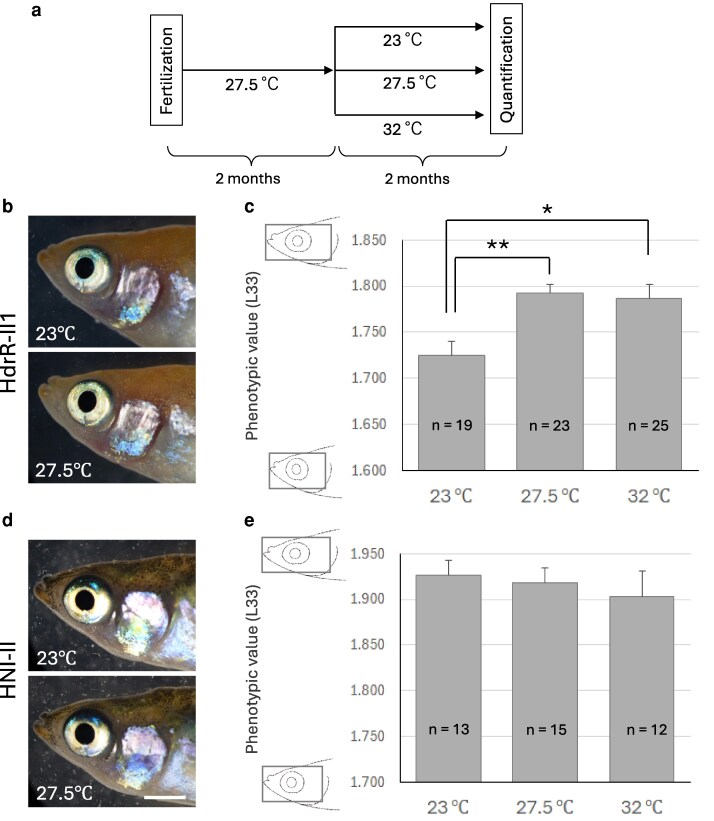
Effects of rearing temperature. a) Schematic representation of the experiment to examine the effect of rearing temperature. After rearing at 27.5 °C for 2 mpf, the medaka were divided into 3 groups and reared for 2 more months at 23 °C, 27.5 °C, and 32 °C, respectively before quantification. b) HdrR-II1 medaka bred at 23 °C (upper) and 27.5 °C (lower) from 2 to 4 mpf. c) Phenotypic values (L33) of the HdrR-II1 medaka reared at 23 °C, 27.5 °C, and 32 °C. Asterisks indicate significant differences (Welch's *t*-test, Bonferroni adjusted *P* < 0.017). *P < 0.01, **P < 0.001. Error bars, S. E. d) HNI-II medaka reared at 23 °C (upper) and 27.5 °C (lower) from 2 to 4 mpf. Bar, 3 mm. e) Phenotypic values (L33) of the HNI-II medaka reared at 23 °C, 27.5 °C, and 32 °C. Error bars, S. E.

During the course of the study, a part of HdrR-II1 medaka were transferred to an outdoor breeding facility at 2 mpf for maintenance due to limited space of in-house facility. Unexpectedly, we noticed that these fish maintained outdoor exhibited intra-strain differences in head proportions (the head length-to-height ratio) that appeared to correlate with seasonal temperature fluctuations. This observation suggested that L33 might retain plasticity in response to environmental temperature even at young-fish stage (2 mpf). To verify this finding, we conducted the experiment shown in Fig. 5a. After rearing HdrR-II1 medaka at 27.5 °C until 2 mpf, we divided the population into 3 groups and reared them at 23 °C, 27.5 °C, and 32 °C, respectively, for an additional 2 months until they reached 4 mpf. We then performed quantitative analysis on these medaka from each temperature group.

The mean L33 values were 1.725 ± 0.015 at 23 °C, 1.793 ± 0.009 at 27.5 °C, and 1.787 ± 0.015 at 32 °C, indicating that rearing at 23 °C significantly reduced the phenotypic value (Fig. 5b and c). No significant difference was observed between the 27.5 °C and 32 °C groups. Consequently, low-temperature rearing resulted in a reduction of ∼0.07 in the mean L33 value—a change comparable to that observed in the C6C strain (Fig. 2a)—which reflects a shorter head length relative to head height. For trait D23, the phenotypic values at 23 °C, 27.5 °C, and 32 °C were 1.094 ± 0.012, 1.104 ± 0.010, and 1.101 ± 0.008, respectively, and no significant differences were observed.

Next, we investigated whether the change in L33 values observed in the HdrR-II1 strain would also occur in other medaka strains. We conducted the same experiment on the HNI-II strain, and as shown in Fig. 5d and e, no difference in phenotypic values was detected among the different temperature conditions. This clearly demonstrates that the observed change in phenotypic value resulting from 2 months of low-temperature rearing is dependent on the genetic background.

## Discussion

Here, we identified an ∼3.5 Mbp region on Chr 6 associated with trait L33, defined as the ratio of head length to head height. Detailed analysis of congenic strains indicated that this region likely contains at least 3 QTLs, designated QTL6-1, QTL6-2, and QTL6-3. These loci do not act as a single QTL contributing independently to the phenotype; rather, their effects depend on specific genetic contexts. Specifically, the phenotypic value decreased when both QTL6-1 and QTL6-2 originated from HNI-II. In addition, the HNI-II-derived QTL6-3 significantly reduced the phenotypic value only when QTL6-1 and QTL6-2 shared the same origin, being either both from HNI-II or both from HdrR-II1 (Fig. 2c). Together, these observations suggest potential genetic interactions, one of which involves between QTL6-3 and the QTL6-1/6-2 region. Notably, Hi-C contact maps revealed physical interactions between the QTL6-3 region and the genomic interval encompassing QTL6-1 and QTL6-2 (Fig. 4). Although chromatin contacts can change during cell differentiation, these interactions were consistently observed in HdrR-II1–derived fibroblasts (Fig. 4) as well as in embryos of both strains (Supplementary Fig. 6), suggesting that similar chromatin associations may also occur in cell lineages contributing to craniofacial structures. Furthermore, the similarity of these contact patterns between the HdrR-II1 and HNI-II strains suggests that the phenotypic differences are likely not caused by large-scale rearrangements of the 3D chromatin layout itself. Instead, this conserved chromatin architecture may provide a stable structural framework that facilitates the combinatorial effects of the 3 linked QTLs. Together, both intra-TAD (QTL6-2 and QTL6-3) and longer-range (QTL6-3 toward QTL6-1 and QTL6-2) chromatin interactions may contribute to variation in the L33 phenotype.

Although our 3-QTL model is a plausible hypothesis, its validation remains technically challenging. To isolate the effects of individual QTLs, we need congenic strains where only each QTL region is replaced with the HNI-II genome. However, it is difficult to generate such congenic strains for relatively small genomic regions (hundreds of kbp to a few Mbp) using conventional or advanced breeding methods (Markel et al. 1997; Shinya et al. 2023), while these regions remain too large to be targeted by current genome-editing approaches (Li et al. 2024). Thus, testing this hypothesis requires new technologies capable of precisely replacing targeted genomic regions of several hundred kbp.

The 3 candidate QTL regions on Chr 6 harbor numerous genes (∼35 genes for QTL6-1, ∼5 for QTL6-2, and ∼28 for QTL6-3) with extensive sequence variations observed between the HdrR-II1 and HNI-II genomes, both within and surrounding these genes (Supplementary Table 8 and Supplementary Figs. 3, 4). Given the proposed genetic interactions between these QTLs, we interrogated the STRING database (https://string-db.org/), which catalogs known and predicted protein-protein interactions, but found no compelling candidate gene sets; although a interaction was detected between *nucleoporin 93* (*nup93*) within QTL6-1 and *nucleoporin 205* (*nup205*) within QTL6-3, the relevance to craniofacial morphology appeared weak. A notable gene is *fgf6*, located within QTL6-2, which is involved in muscle tissue development and regeneration (Armand et al. 2006). Sequence variations identified in its proximal upstream region and an intron (Supplementary Fig. 5) may alter transcriptional or post-transcriptional regulation, potentially driving the observed phenotypic divergence. However, functional validation of *fgf6* and its interaction with QTL6-1 remains for future studies.

Our study revealed that both L33 and D23 are influenced by multiple QTLs situated on a single chromosome (Chr 6 and Chr 15, respectively, Figs. 2 and 3). In particular, the QTLs for L33 are clustered within an exceptionally narrow genomic interval of ∼2 Mbp (Fig. 2b). Studies on human craniofacial morphology also suggest the involvement of numerous loci (Selleri and Rijli 2023), often found in relatively close proximity on the same chromosome (Pickrell et al. 2016; Claes et al. 2018; Xiong et al. 2019). Interestingly, the colocalization of multiple QTLs within restricted genomic regions has been documented in several studies across a wide range of traits, including body size (Christians and Keightley 2004; Christians et al. 2006), obesity (Stewart et al. 2012), atherosclerotic phenotype (Ghazalpour et al. 2006), and behavior (Kato et al. 2014; Takahashi et al. 2015). Collectively, existing studies and our findings suggest that such clustering is a recurrent pattern in the genetic architecture of complex traits across species. This spatial clustering raises the possibility that functional interactions among these loci are mediated by physical chromatin contacts, highlighting the importance of chromatin conformation analyses.

Our study also revealed that 2 months of low-temperature rearing induced a reduction of ∼0.07 in the mean L33 value (Fig. 5c)—a change comparable in magnitude to the genetic contribution of the 6 Mbp HNI-II-derived region in the C6C congenic strain (Fig. 2a). Notably, this environmental effect was observed in HdrR-II1 but was absent in HNI-II, indicating that the response to low temperature is contingent upon the genetic background. Consequently, these findings establish the L33 trait as a good model for the experimental analysis of genotype × environment (G × E) interactions.

Despite the recognized importance of environmental factors, most craniofacial research remains correlational, such as the links between human cranial indices and climatic variables (Perez and Monteiro 2009; Pan et al. 2014; Matsumura et al. 2024). To our knowledge, only a few experimental studies exist, each with notable limitations: for instance, research in zebrafish focused primarily on overall body shape with only limited craniofacial analysis (Georga and Koumoundouros 2010), while rat studies utilized extreme 17 °C temperature differences (Steegmann and Platner 1968; Rae et al. 2006). In contrast, we observed significant phenotypic shifts in medaka in response to a subtle 4.5 °C difference within their normal physiological range. This high sensitivity to temperature change suggests that medaka possess a sophisticated mechanism for translating subtle thermal fluctuations into phenotypic shifts. This feature makes medaka a useful model system for investigating how ambient environments shape craniofacial morphology.

In conclusion, we identified the craniofacial trait in medaka that is influenced by both genetic factors and environmental temperature. This system provides a powerful model for understanding how these 2 fundamental determinants interact to generate individual phenotypic diversity. To gain molecular insights into this process, it will be essential to determine when and how specific cell lineages contribute to L33 craniofacial morphology, and to perform gene expression analyses, functional characterization of candidate genes, and Hi-C–based chromatin interaction analyses within these lineages.

## Supplementary Material

jkag094_Supplementary_Data

## Data Availability

All congenic strains but HdrR-II1-Chr15G^HNI-II (C15G) generated in this study are available from NBRP Medaka (https://shigen.nig.ac.jp/medaka/). C15G could not be maintained because of severe occurrence of sex reversal. Hi-C datasets are available from DDBJ under BioProject accession number PRJDB7492 and PRJDB19938. All other data including phenotype data of F_2_ progeny for QTL mapping, are present within the article, figures, tables and supplementary files. Supplementary Tables are included in Supplementary File 1, Supplementary Figs. 1, 2, 5, 6 are in Supplementary File 2, and Supplementary Figs. 3, 4 are in Supplementary File 3. Supplemental material available at *G3* online.
