# Supplementary material for: Linked genetic loci and genotype-dependent temperature effects shape craniofacial morphology in medaka
Source: G3 (Bethesda). 2026 Apr 9;16(6):jkag094. doi: 10.1093/g3journal/jkag094 (PMC13232513; doi:10.1093/g3journal/jkag094)
Supplement: jkag094_Supplementary_Data [file jkag094_supplementary_data.zip › Supplementary_File_2_G3-2026-406592.pdf]

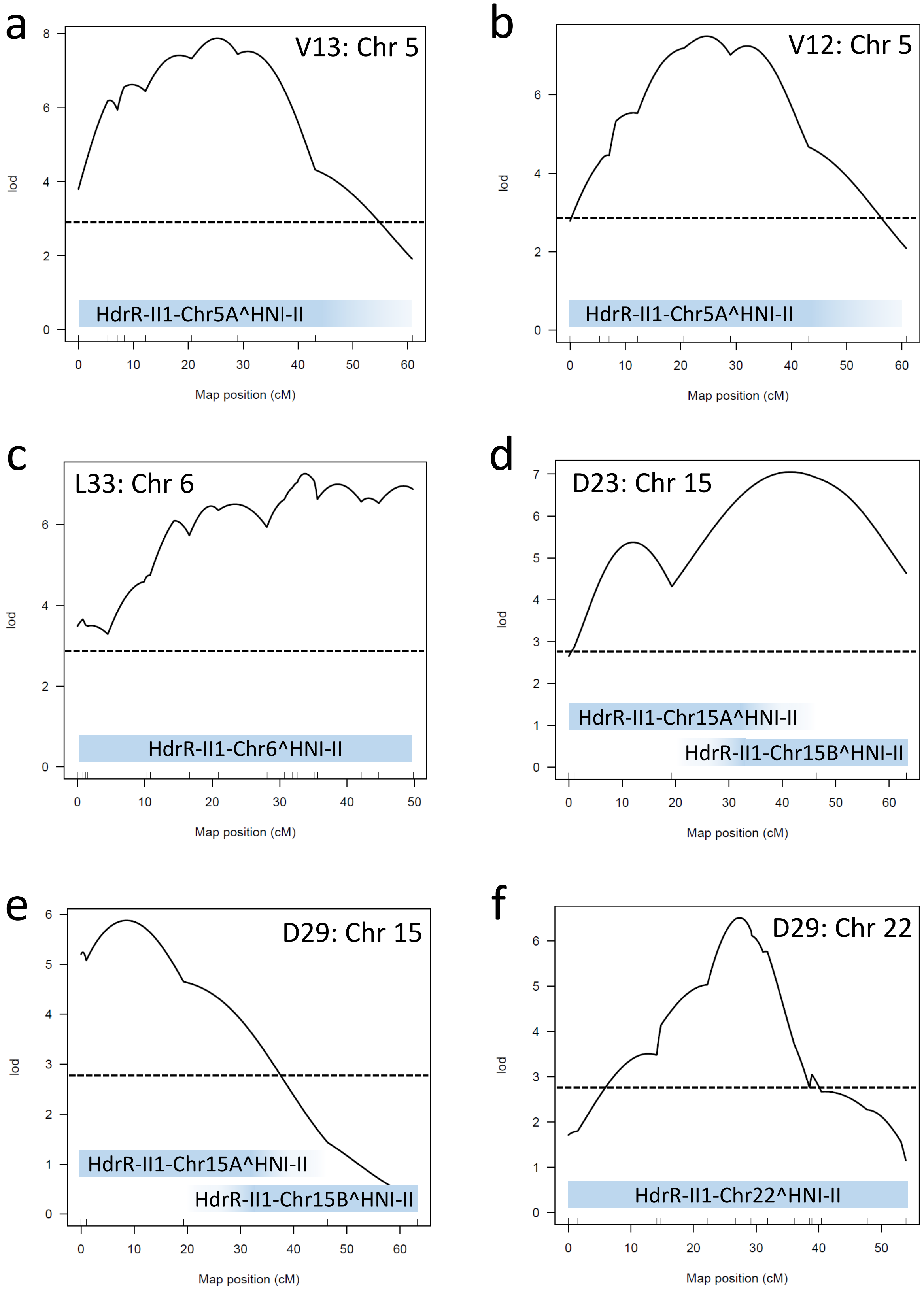

Supplementary Fig. 1

QTLs selected for validation. (a) QTL associated with V13 on Chr 5. (b) QTL associated with V12 on Chr 5. (c) QTL associated with L33 on Chr 6. (d) QTL associated with D23 on Chr 15. (e) QTL associated with D29 on Chr 15. (f) QTL associated with D29 on Chr 22. The dashed line indicates the significance of LOD threshold. Chromosomal regions that were substituted with DNA from HNI-II in congenic/consomic strains for validation are shown in blue.

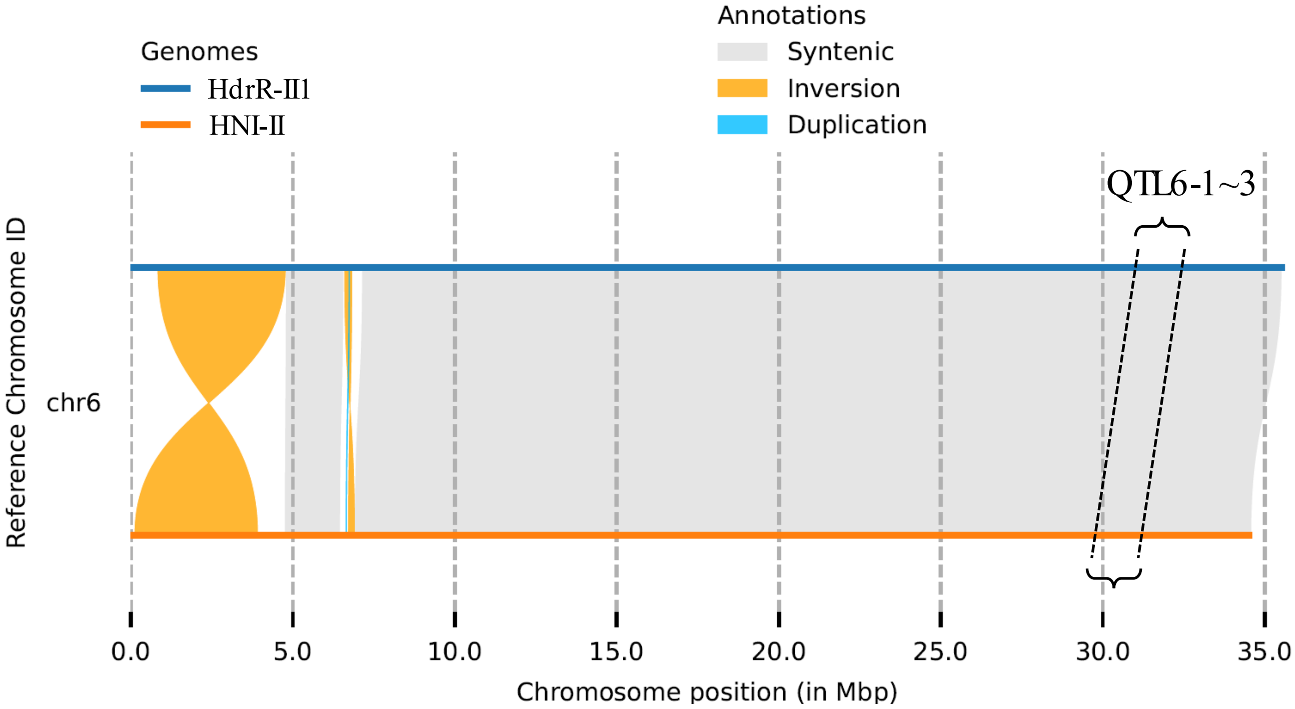

Supplementary Fig. 2

Genomic synteny and rearrangements on Chr 6 between the HdrR-II1 and HNI-II. The HNI-II and the HdrR-II1 genome are represented by blue and orange lines, respectively. Gray area indicates syntenic region, orange area indicates inverted region, and blue area indicates duplicated region.

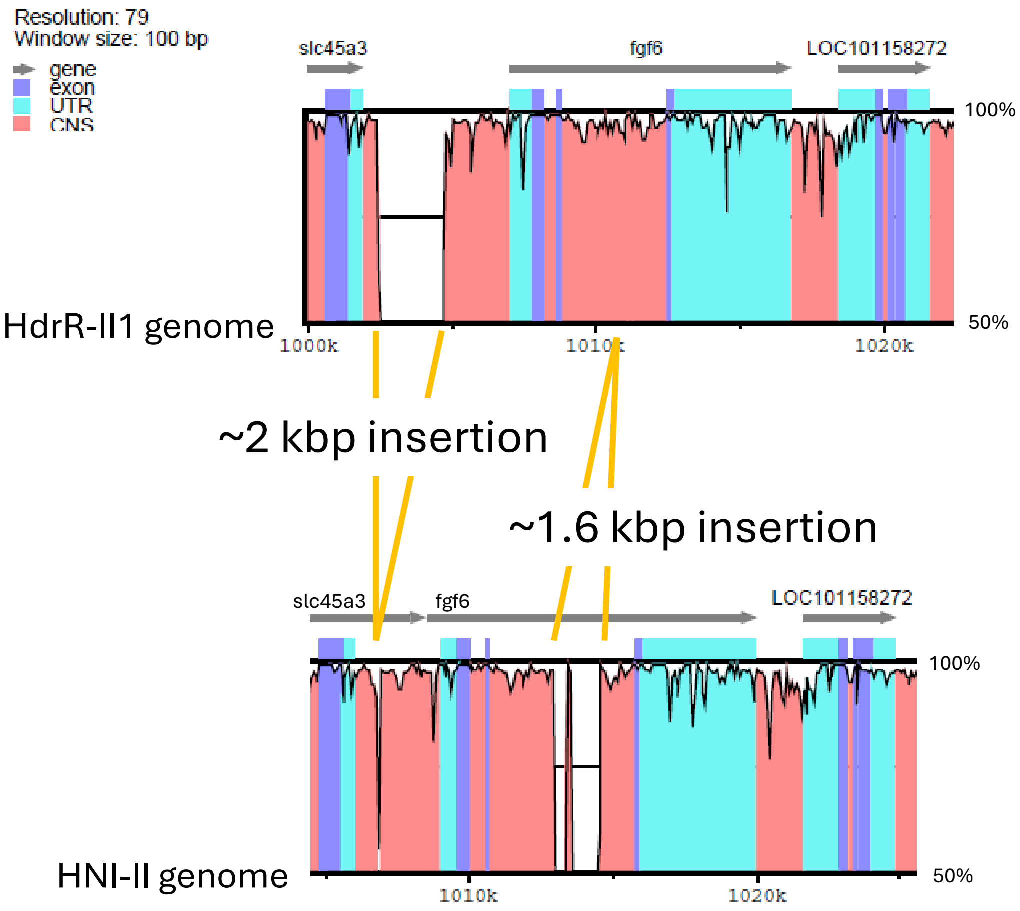

Supplementary Fig. 5

VISTA plot around *fgf6.* The HNI-II genome aligned to the HdrR-II1 (top) and the HdrR-II1 genome aligned to the HNI-II (bottom). A ~2 kbp insertion was found approximately 2 kbp upstream of *fgf6* in the HdrR-II1 genome, and a ~1.6 kbp insertion was found within the second intron in the HNI-II genome. The level of conservation (vertical axis) is shown in the coordinates of the referenced sequence (horizontal axis). Conserved regions above the level of 70% per 100 bp are highlighted under the curve, with red indicating a conserved non-coding region, violet, a conserved exon, and blue, an untranslated region. Genes are represented by gray arrows.

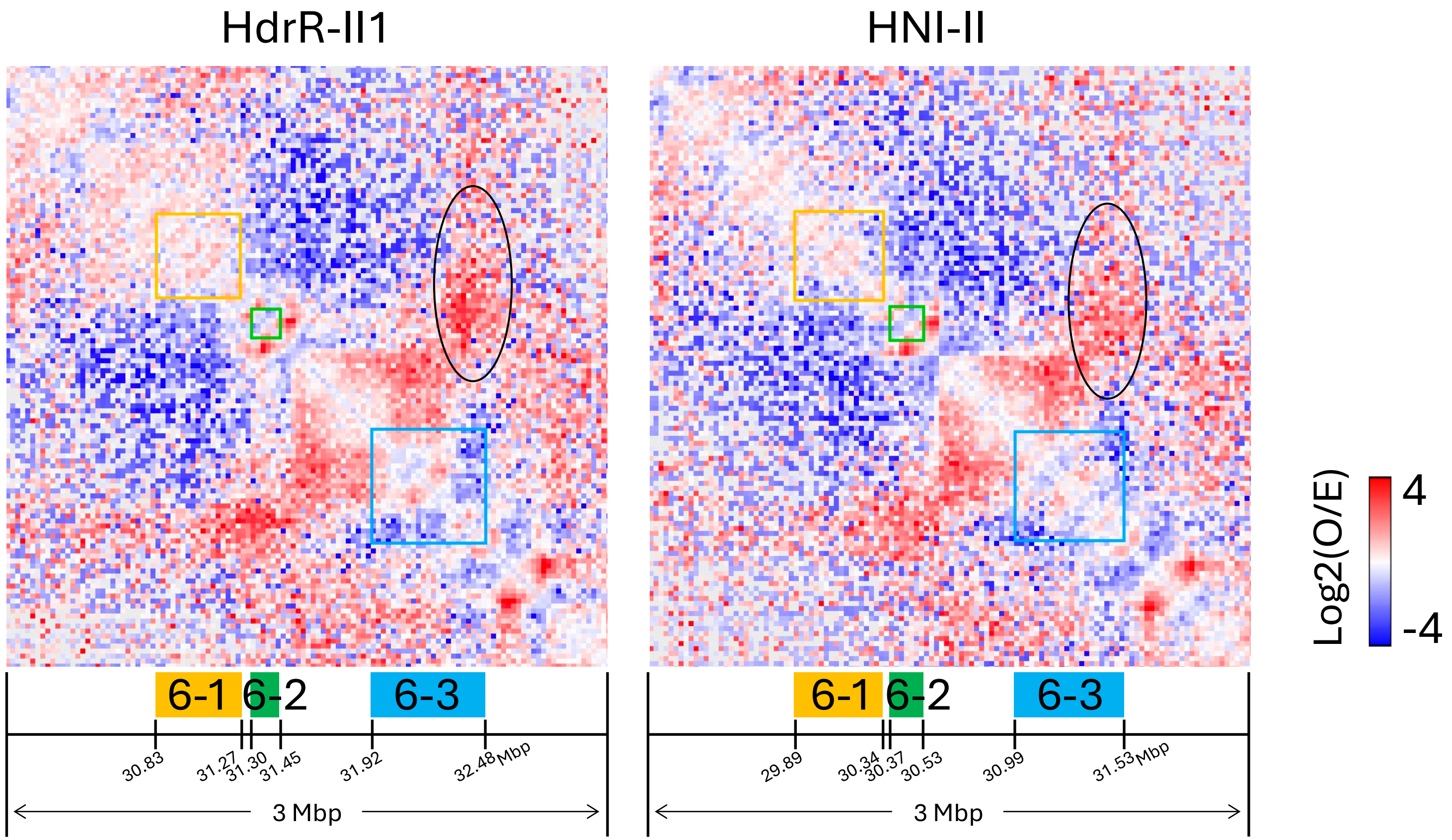

Supplementary Fig. 6

Observed/expected (O/E) contact map of 6-somite stage embryos from HdrR-II1 (left) and HNI-II (right) at 25-kb resolution. The regions of three candidate QTLs are squared: QTL6-1 (yellow), QTL6-2 (green) and QTL6-3 (blue). Arrows indicate the long-range contact.
