# Supplementary material for: Linked genetic loci and genotype-dependent temperature effects shape craniofacial morphology in medaka
Source: G3 (Bethesda). 2026 Apr 9;16(6):jkag094. doi: 10.1093/g3journal/jkag094 (PMC13232513; doi:10.1093/g3journal/jkag094)
Supplement: jkag094_Supplementary_Data [file jkag094_supplementary_data.zip › Supplementary_File_3_G3-2026-406592.pdf]

Supplementary Fig. 3

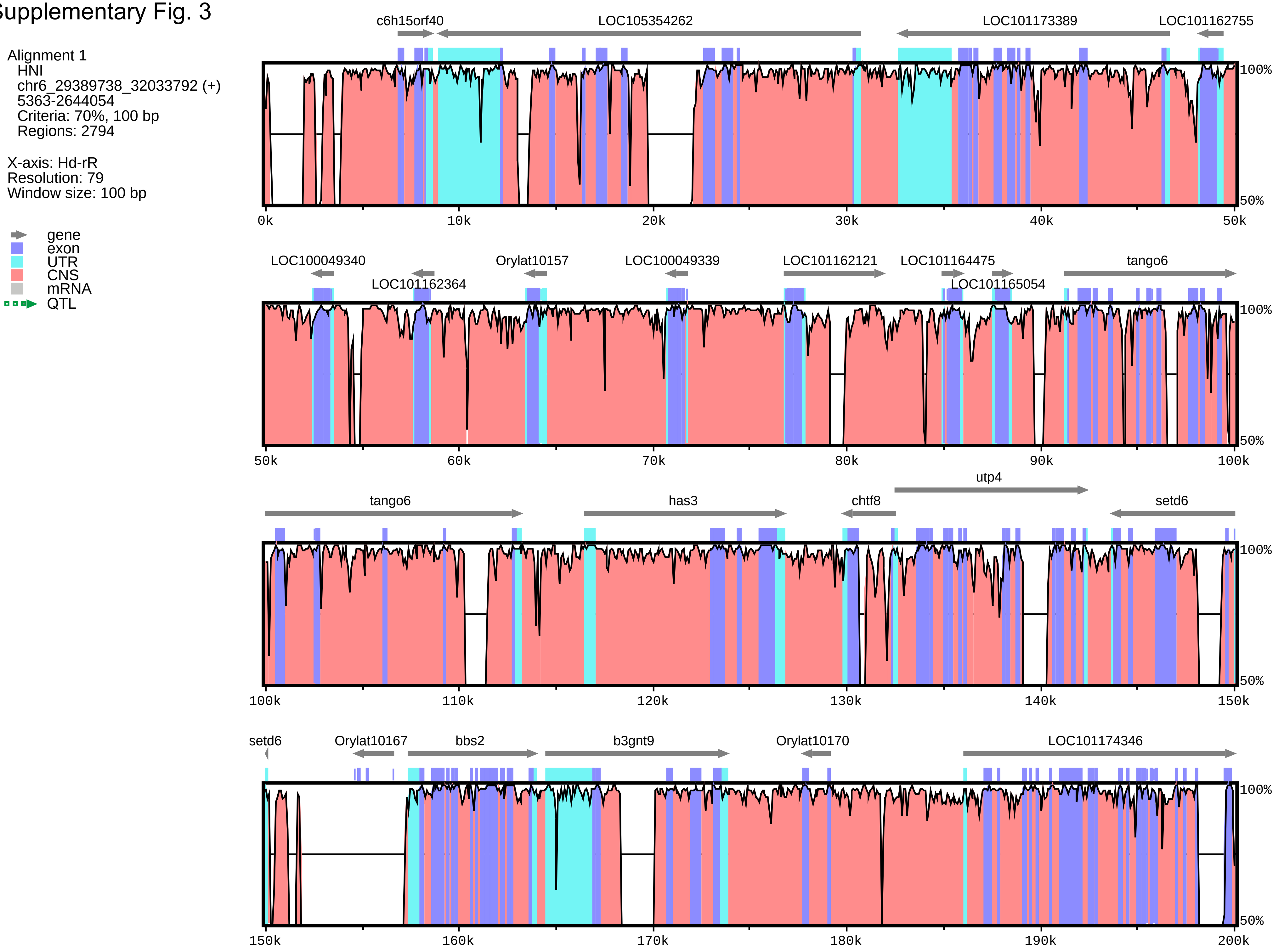

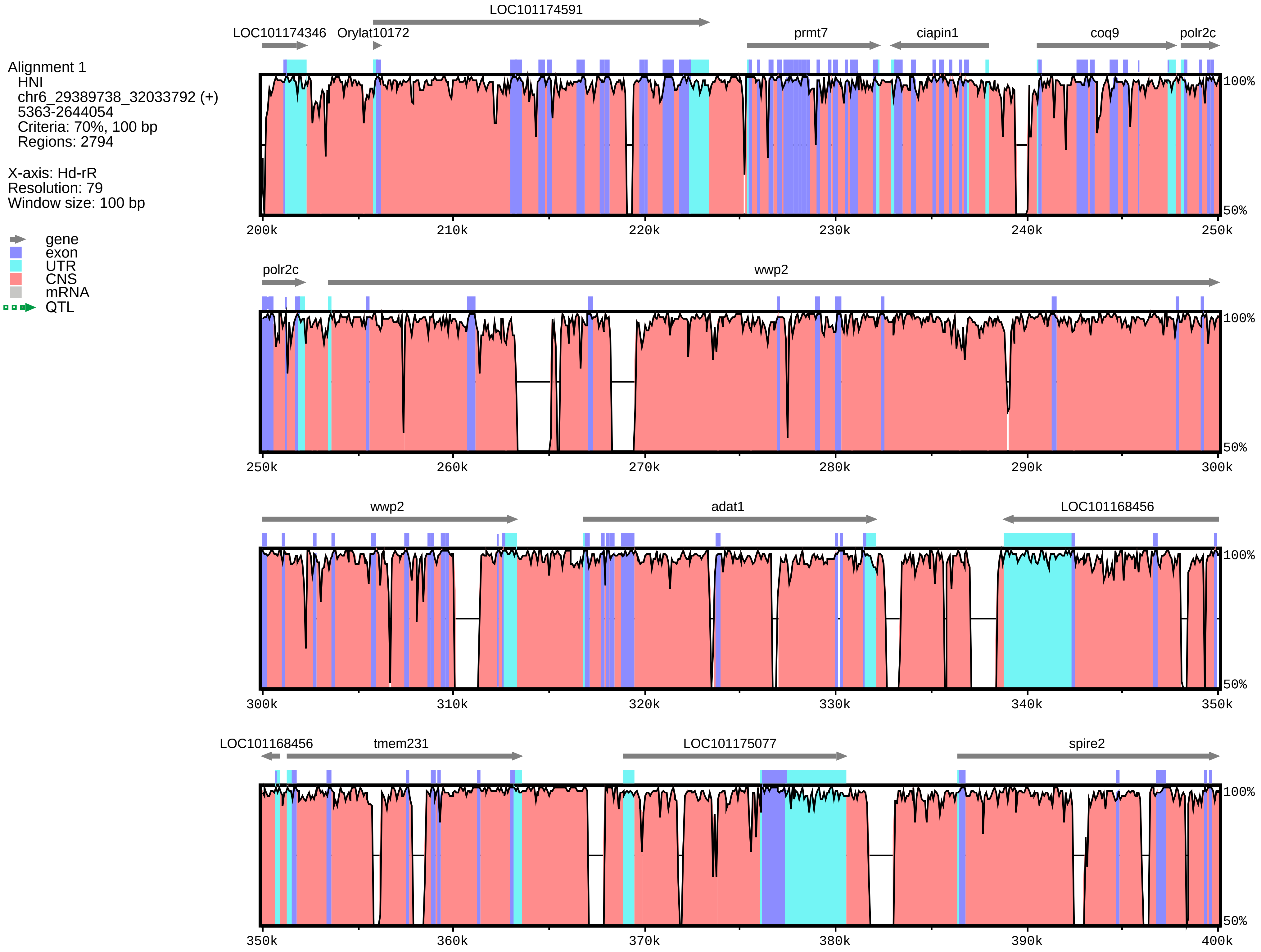
Alignment 1
HNI
chr6_29389738_32033792 (+)
5363-2644054
Criteria: 70%, 100 bp
Regions: 2794
X-axis: Hd-rR
Resolution: 79
Window size: 100 bp
gene
exon
UTR
CNS
mRNA
QTL
LOC101174591
LOC101174346
Orylat10172
prmt7
ciapin1
coq9
polr2c
100%
50%
200k
210k
220k
230k
240k
250k
polr2c
wwp2
250k
260k
270k
280k
290k
300k
wwp2
adat1
LOC101168456
300k
310k
320k
330k
340k
350k
LOC101168456
tmem231
LOC101175077
spire2
350k
360k
370k
380k
390k
400k

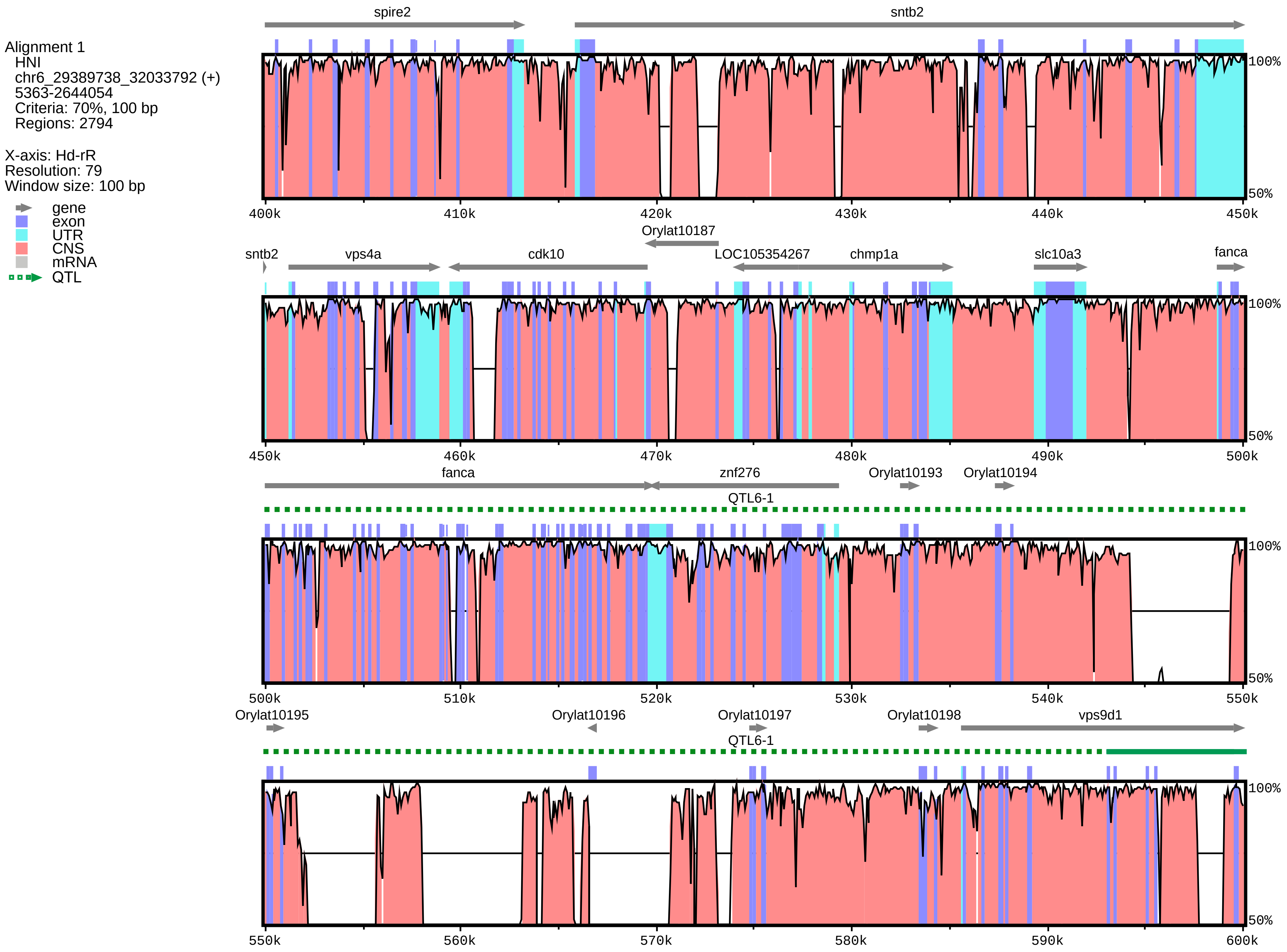

Alignment 1
HNI
chr6_29389738_32033792 (+)
5363-2644054
Criteria: 70%, 100 bp
Regions: 2794
X-axis: Hd-rR
Resolution: 79
Window size: 100 bp
gene
exon
UTR
CNS
mRNA
QTL
spire2
sntb2
100%
50%
400k
410k
420k
430k
440k
450k
Orylat10187
sntb2
vps4a
cdk10
LOC105354267
chmp1a
slc10a3
fanca
460k
470k
480k
490k
500k
fanca
znf276
Orylat10193
Orylat10194
QTL6-1
510k
520k
530k
540k
550k
Orylat10195
Orylat10196
Orylat10197
Orylat10198
vps9d1
QTL6-1
560k
570k
580k
590k
600k

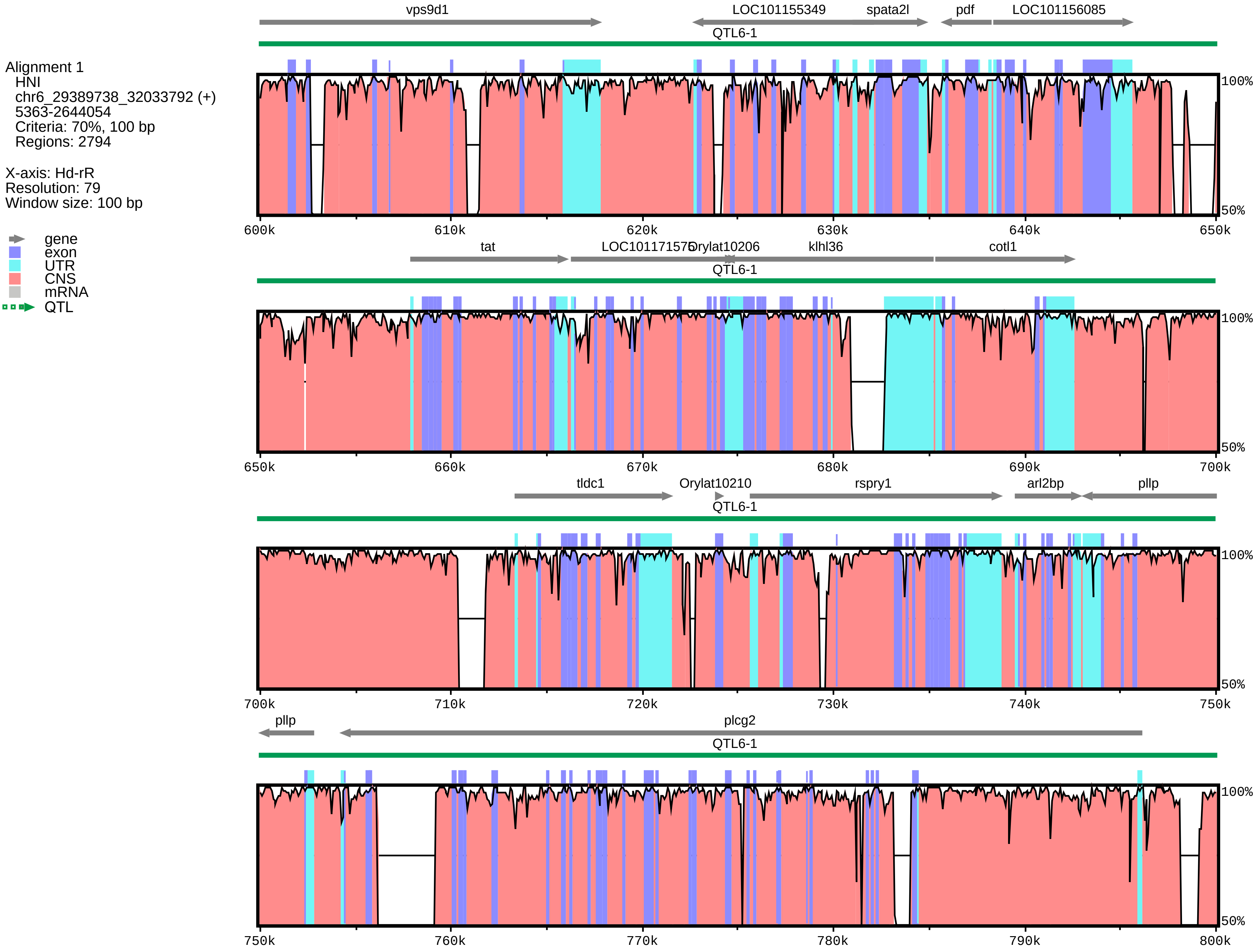

Alignment 1
HNI
chr6_29389738_32033792 (+)
5363-2644054
Criteria: 70%, 100 bp
Regions: 2794
X-axis: Hd-rR
Resolution: 79
Window size: 100 bp
gene
exon
UTR
CNS
mRNA
QTL
vps9d1
LOC101155349
spata2l
pdf
LOC101156085
QTL6-1
100%
50%
600k
610k
620k
630k
640k
650k
tat
LOC101171576
Orylat10206
klhl36
cotl1
660k
670k
680k
690k
700k
tldc1
Orylat10210
rspry1
arl2bp
pllp
710k
720k
730k
740k
750k
plcg2
760k
770k
780k
790k
800k

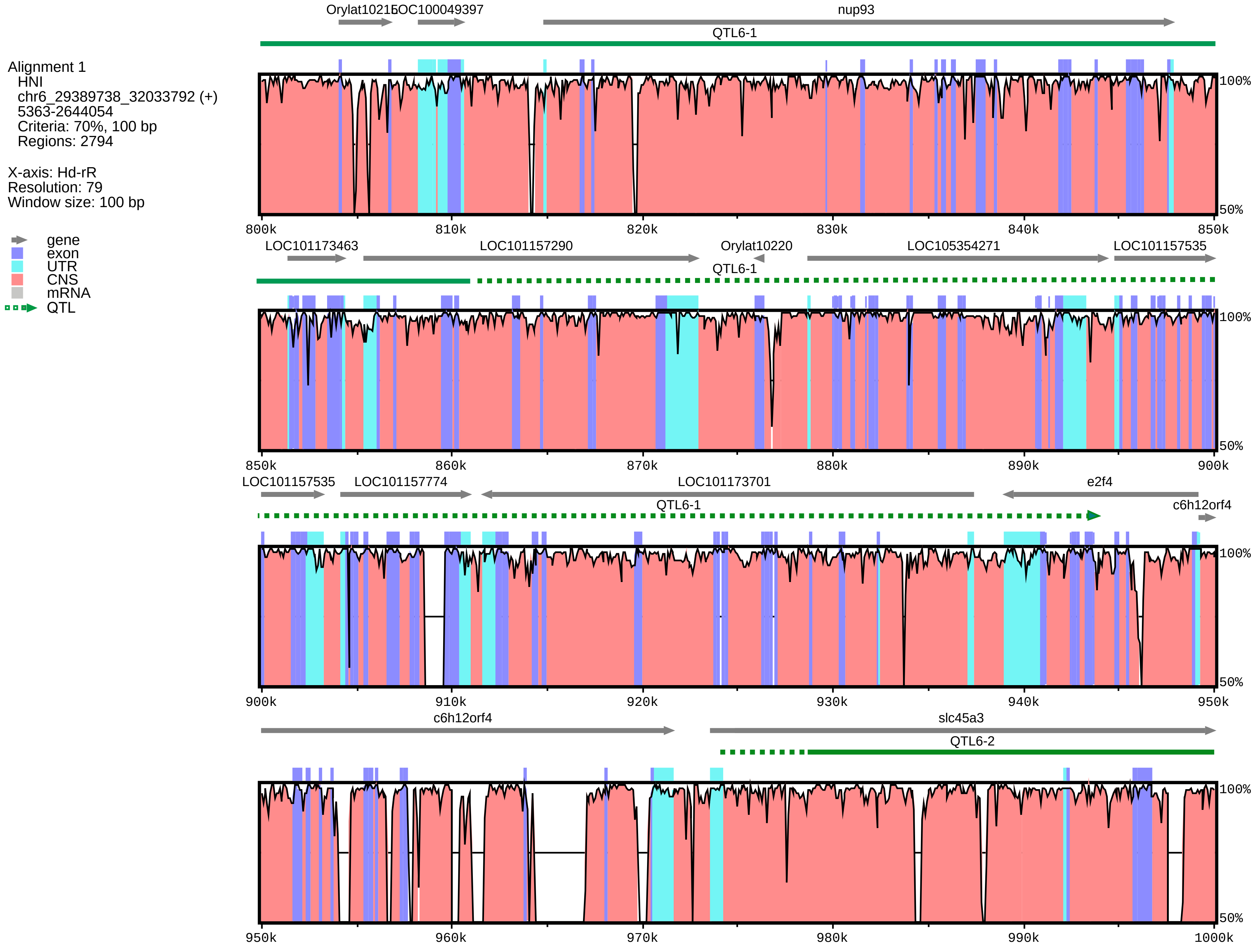

Alignment 1
HNI
chr6_29389738_32033792 (+)
5363-2644054
Criteria: 70%, 100 bp
Regions: 2794
X-axis: Hd-rR
Resolution: 79
Window size: 100 bp
gene
exon
UTR
CNS
mRNA
QTL
Orylat10215
LOC100049397
nup93
QTL6-1
100%
50%
800k
810k
820k
830k
840k
850k
LOC101173463
LOC101157290
Orylat10220
LOC105354271
LOC101157535
QTL6-1
860k
870k
880k
890k
900k
LOC101157535
LOC101157774
LOC101173701
e2f4
c6h12orf4
QTL6-1
910k
920k
930k
940k
950k
c6h12orf4
slc45a3
QTL6-2
960k
970k
980k
990k
1000k

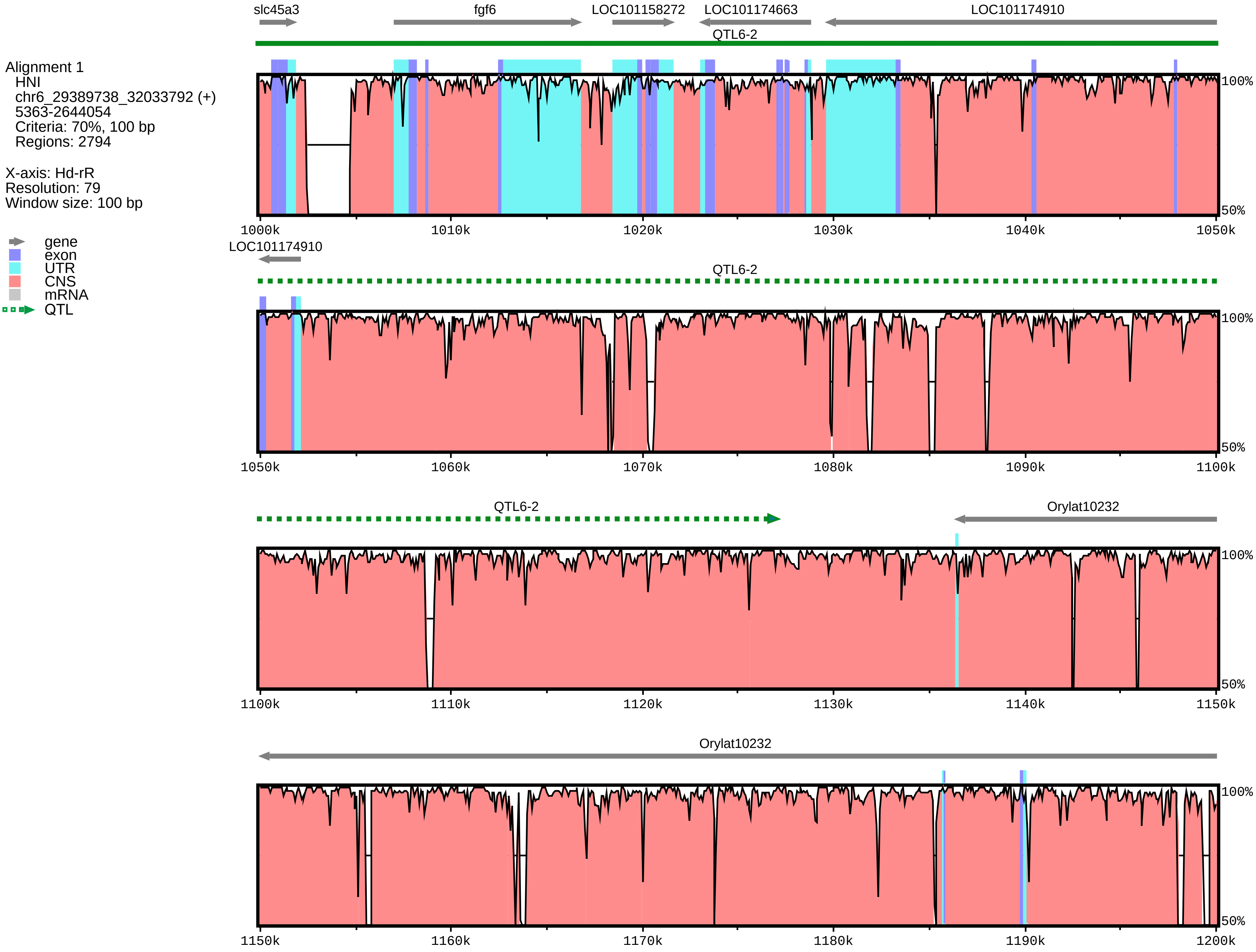

Alignment 1
HNI
chr6_29389738_32033792 (+)
5363-2644054
Criteria: 70%, 100 bp
Regions: 2794
X-axis: Hd-rR
Resolution: 79
Window size: 100 bp
gene
exon
UTR
CNS
mRNA
QTL
slc45a3
fgf6
LOC101158272
LOC101174663
LOC101174910
QTL6-2
Orylat10232
100%
50%
1000k
1010k
1020k
1030k
1040k
1050k
1060k
1070k
1080k
1090k
1100k
1110k
1120k
1130k
1140k
1150k
1160k
1170k
1180k
1190k
1200k

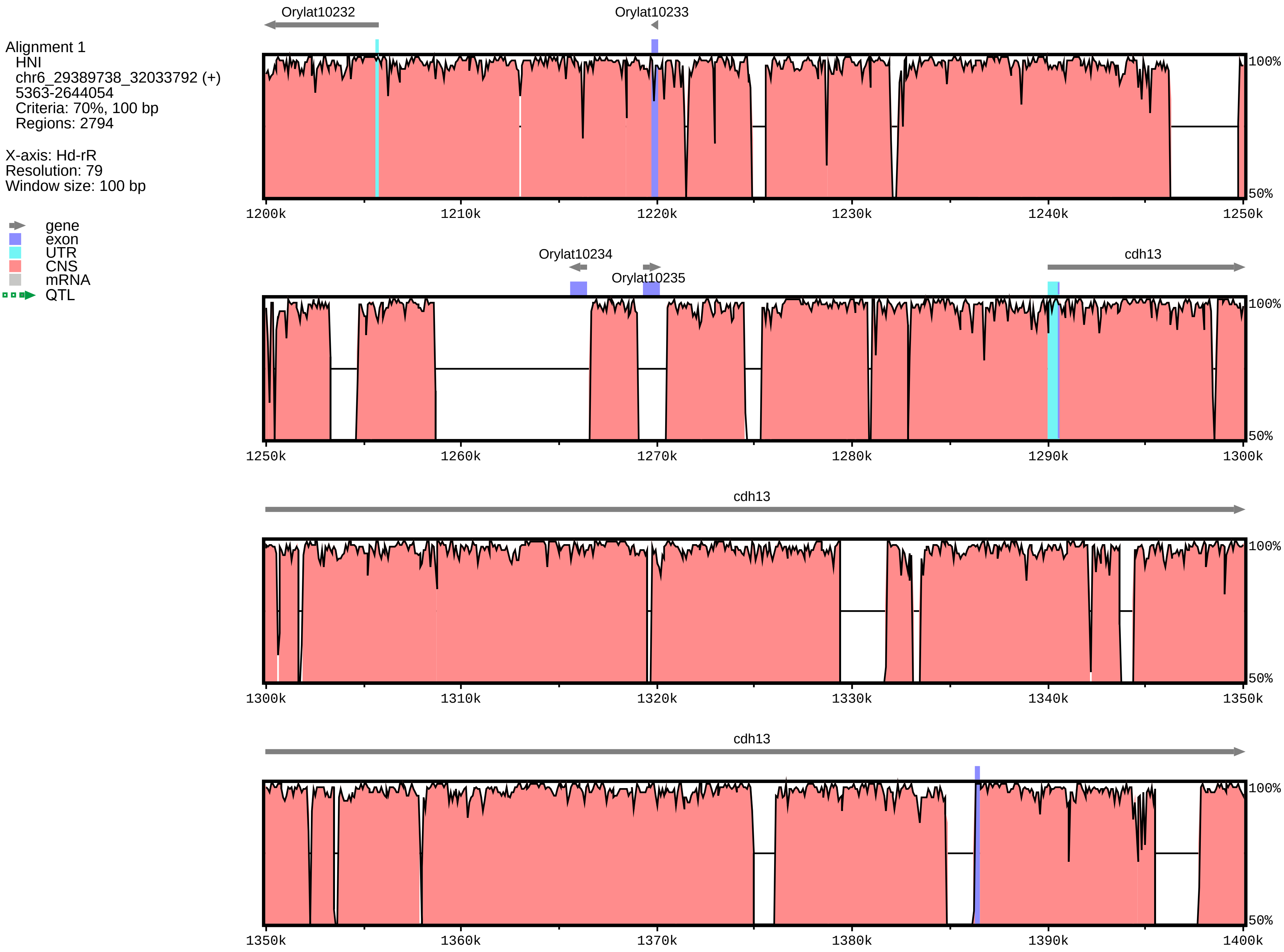
Alignment 1
HNI
chr6_29389738_32033792 (+)
5363-2644054
Criteria: 70%, 100 bp
Regions: 2794
X-axis: Hd-rR
Resolution: 79
Window size: 100 bp
gene
exon
UTR
CNS
mRNA
QTL
Orylat10232
Orylat10233
100%
50%
1200k
1210k
1220k
1230k
1240k
1250k
Orylat10234
Orylat10235
cdh13
1260k
1270k
1280k
1290k
1300k
cdh13
1310k
1320k
1330k
1340k
1350k
cdh13
1360k
1370k
1380k
1390k
1400k

Alignment 1
HNI
chr6_29389738_32033792 (+)
5363-2644054
Criteria: 70%, 100 bp
Regions: 2794

X-axis: Hd-rR
Resolution: 79
Window size: 100 bp

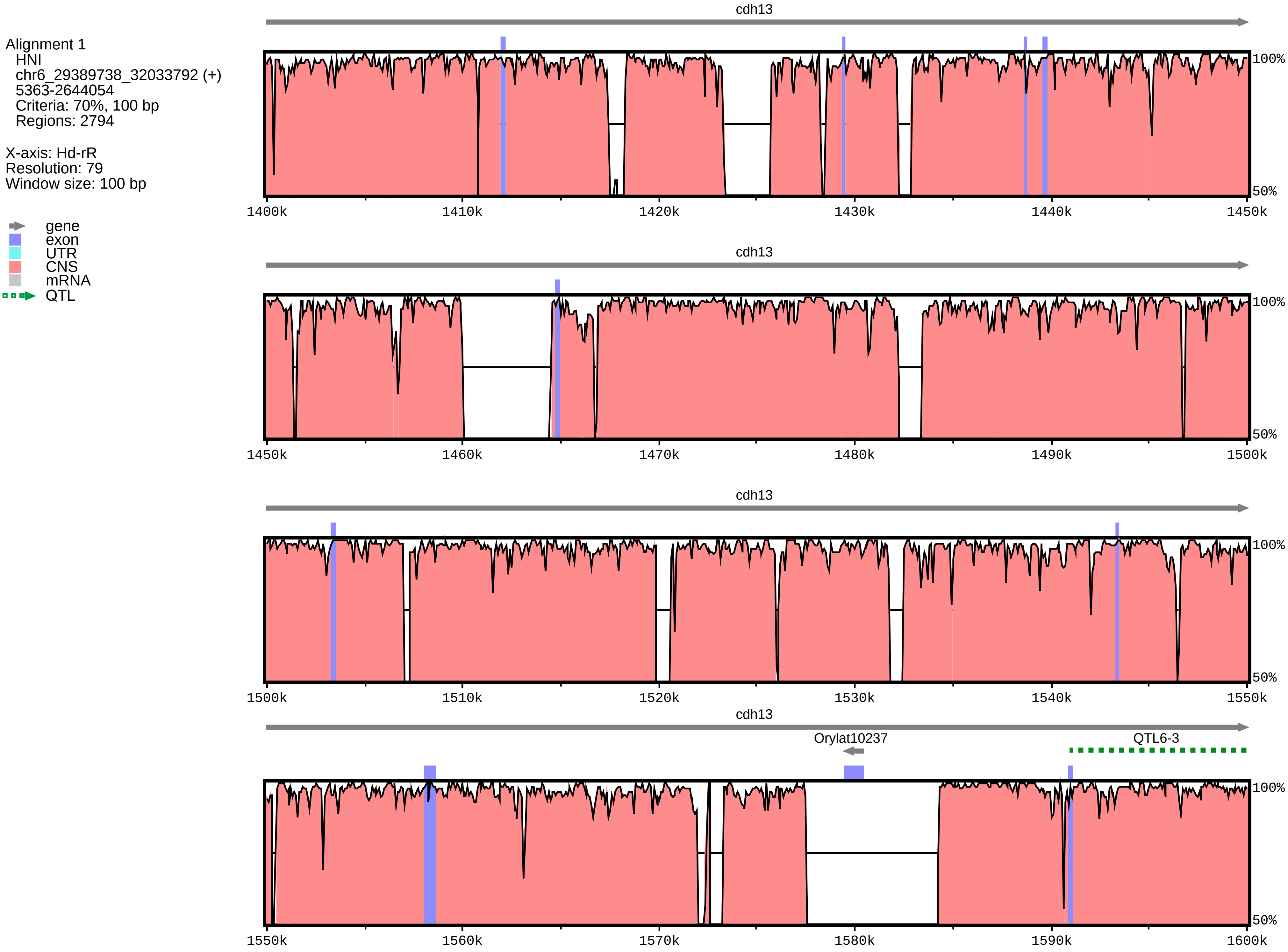

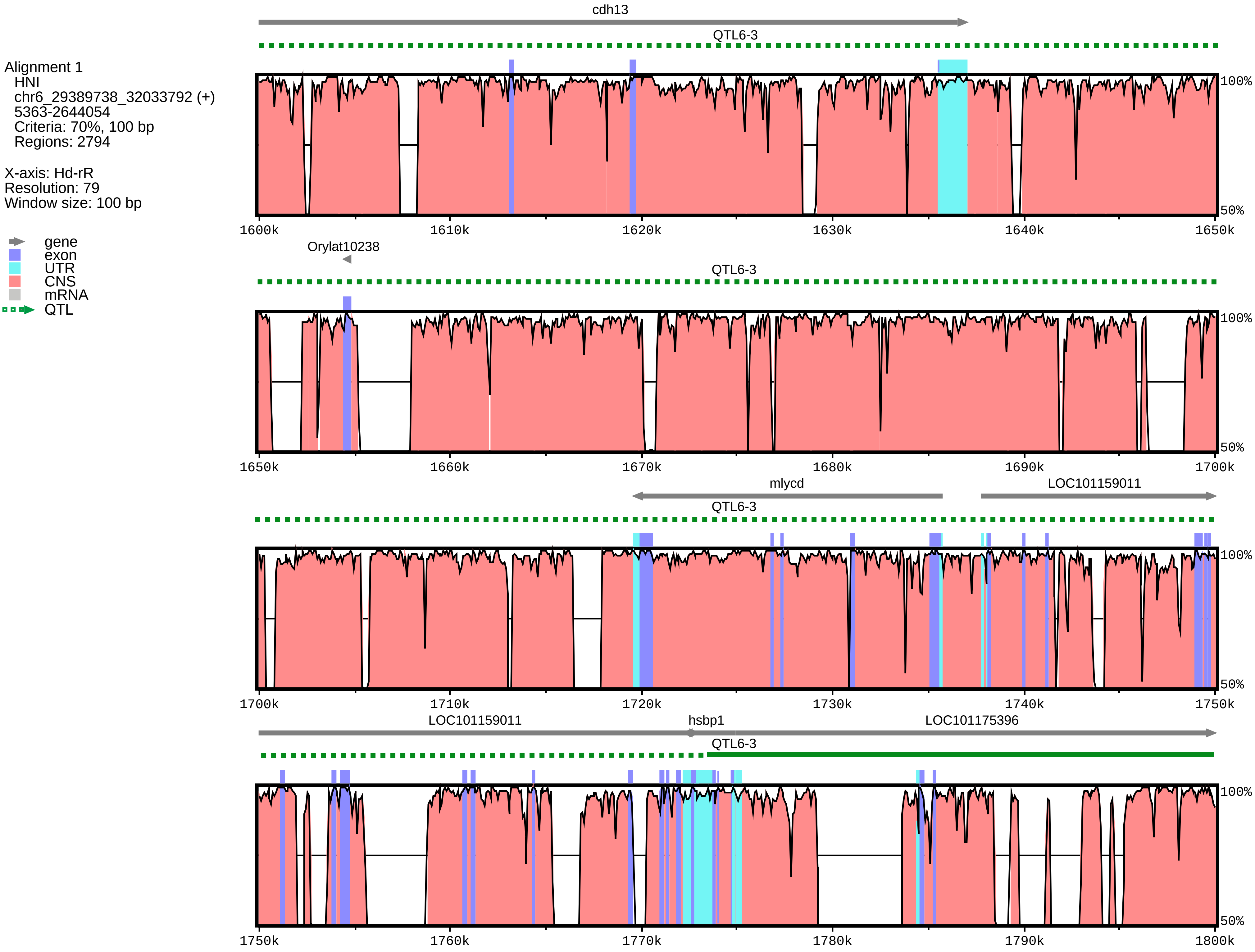

Alignment 1
HNI
chr6_29389738_32033792 (+)
5363-2644054
Criteria: 70%, 100 bp
Regions: 2794
X-axis: Hd-rR
Resolution: 79
Window size: 100 bp
gene
exon
UTR
CNS
mRNA
QTL
cdh13
QTL6-3
100%
50%
1600k
1610k
1620k
1630k
1640k
1650k
Orylat10238
QTL6-3
100%
50%
1650k
1660k
1670k
1680k
1690k
1700k
mlycd
LOC101159011
QTL6-3
100%
50%
1700k
1710k
1720k
1730k
1740k
1750k
LOC101159011
hsbp1
LOC101175396
QTL6-3
100%
50%
1750k
1760k
1770k
1780k
1790k
1800k

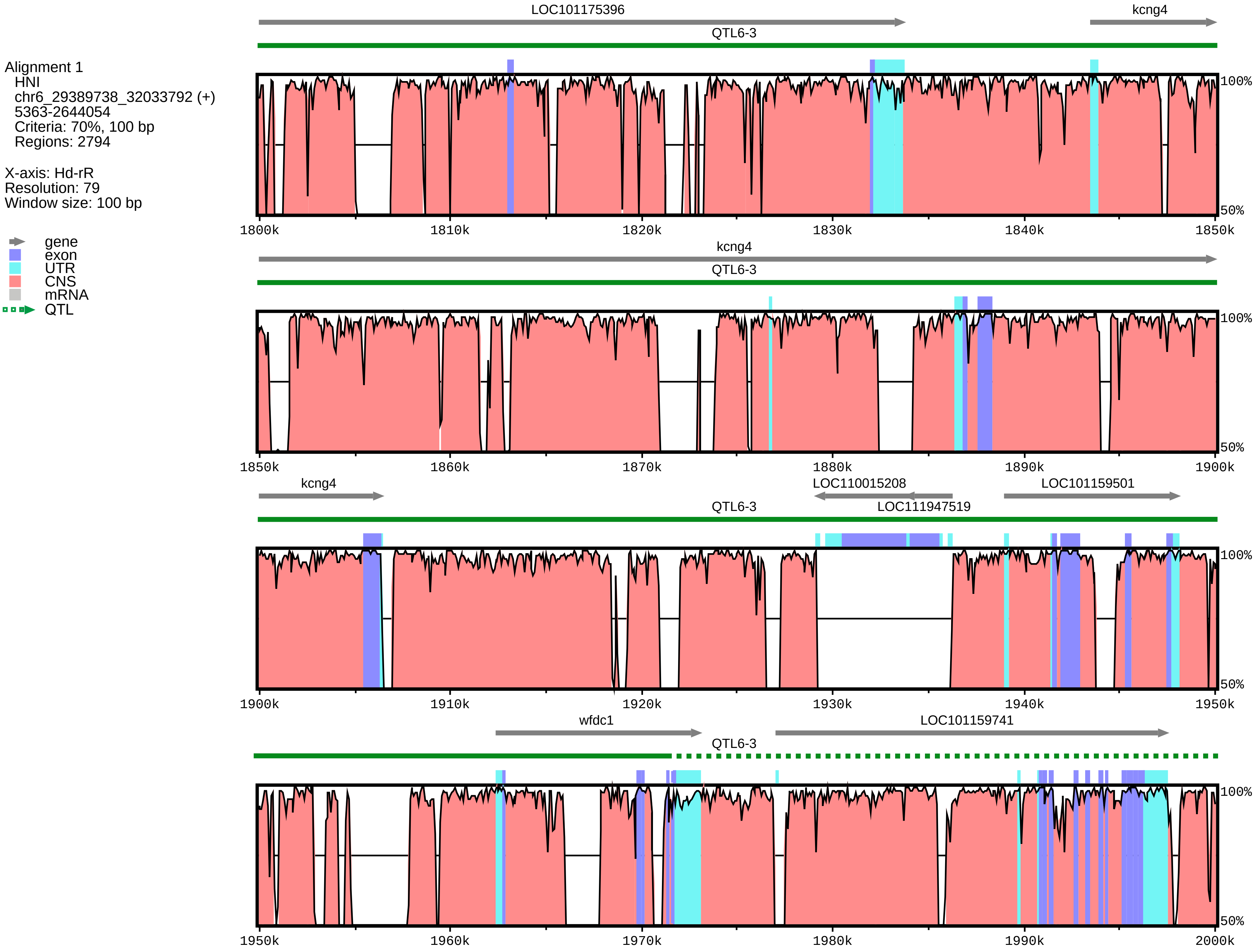
Alignment 1
HNI
chr6_29389738_32033792 (+)
5363-2644054
Criteria: 70%, 100 bp
Regions: 2794
X-axis: Hd-rR
Resolution: 79
Window size: 100 bp
gene
exon
UTR
CNS
mRNA
QTL
LOC101175396
kcng4
QTL6-3
100%
50%
1800k
1810k
1820k
1830k
1840k
1850k
kcng4
QTL6-3
1850k
1860k
1870k
1880k
1890k
1900k
kcng4
LOC110015208
LOC101159501
LOC111947519
QTL6-3
1900k
1910k
1920k
1930k
1940k
1950k
wfdc1
LOC101159741
QTL6-3
1950k
1960k
1970k
1980k
1990k
2000k

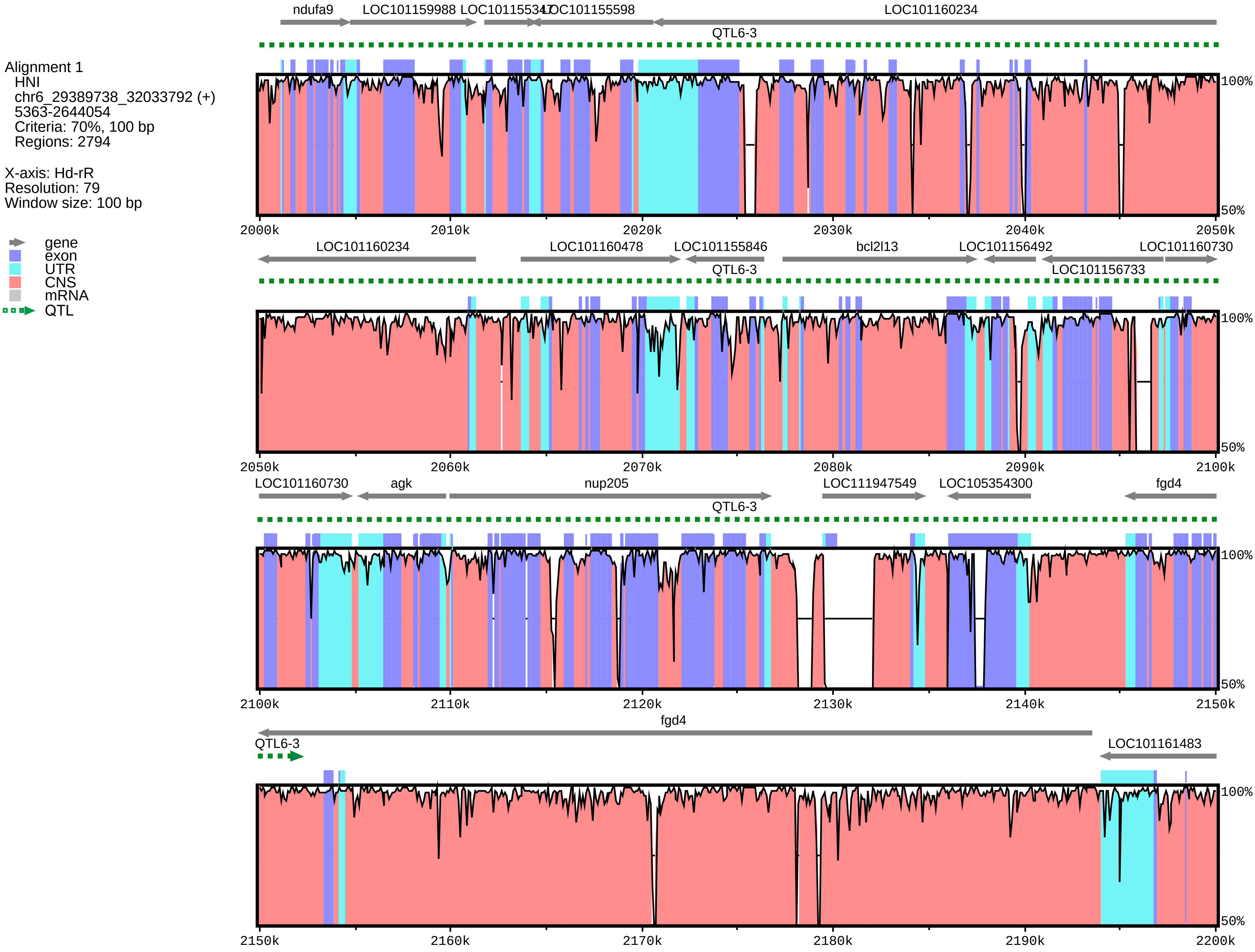
Alignment 1
HNI
chr6_29389738_32033792 (+)
5363-2644054
Criteria: 70%, 100 bp
Regions: 2794
X-axis: Hd-rR
Resolution: 79
Window size: 100 bp
gene
exon
UTR
CNS
mRNA
QTL
ndufa9
LOC101159988
LOC101155347
LOC101155598
LOC101160234
QTL6-3
100%
50%
2000k
2010k
2020k
2030k
2040k
2050k
LOC101160478
LOC101155846
bcl2l13
LOC101156492
LOC101160730
LOC101156733
2060k
2070k
2080k
2090k
2100k
agk
nup205
LOC111947549
LOC105354300
fgd4
2110k
2120k
2130k
2140k
2150k
LOC101161483
2160k
2170k
2180k
2190k
2200k

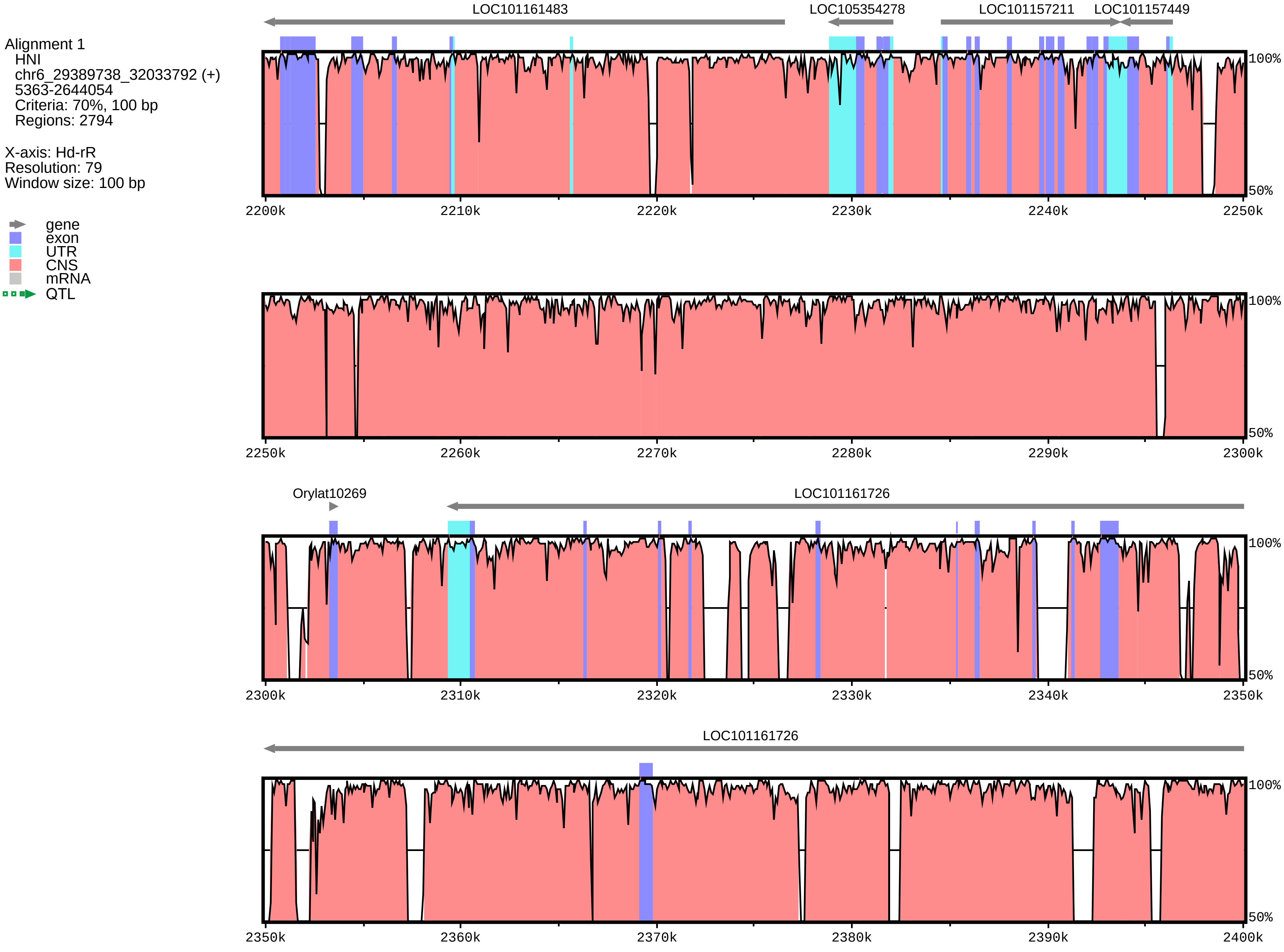
Alignment 1
HNI
chr6_29389738_32033792 (+)
5363-2644054
Criteria: 70%, 100 bp
Regions: 2794
X-axis: Hd-rR
Resolution: 79
Window size: 100 bp
gene
exon
UTR
CNS
mRNA
QTL
LOC101161483
LOC105354278
LOC101157211
LOC101157449
Orylat10269
LOC101161726
LOC101161726
100%
50%
2200k
2210k
2220k
2230k
2240k
2250k
2260k
2270k
2280k
2290k
2300k
2310k
2320k
2330k
2340k
2350k
2360k
2370k
2380k
2390k
2400k

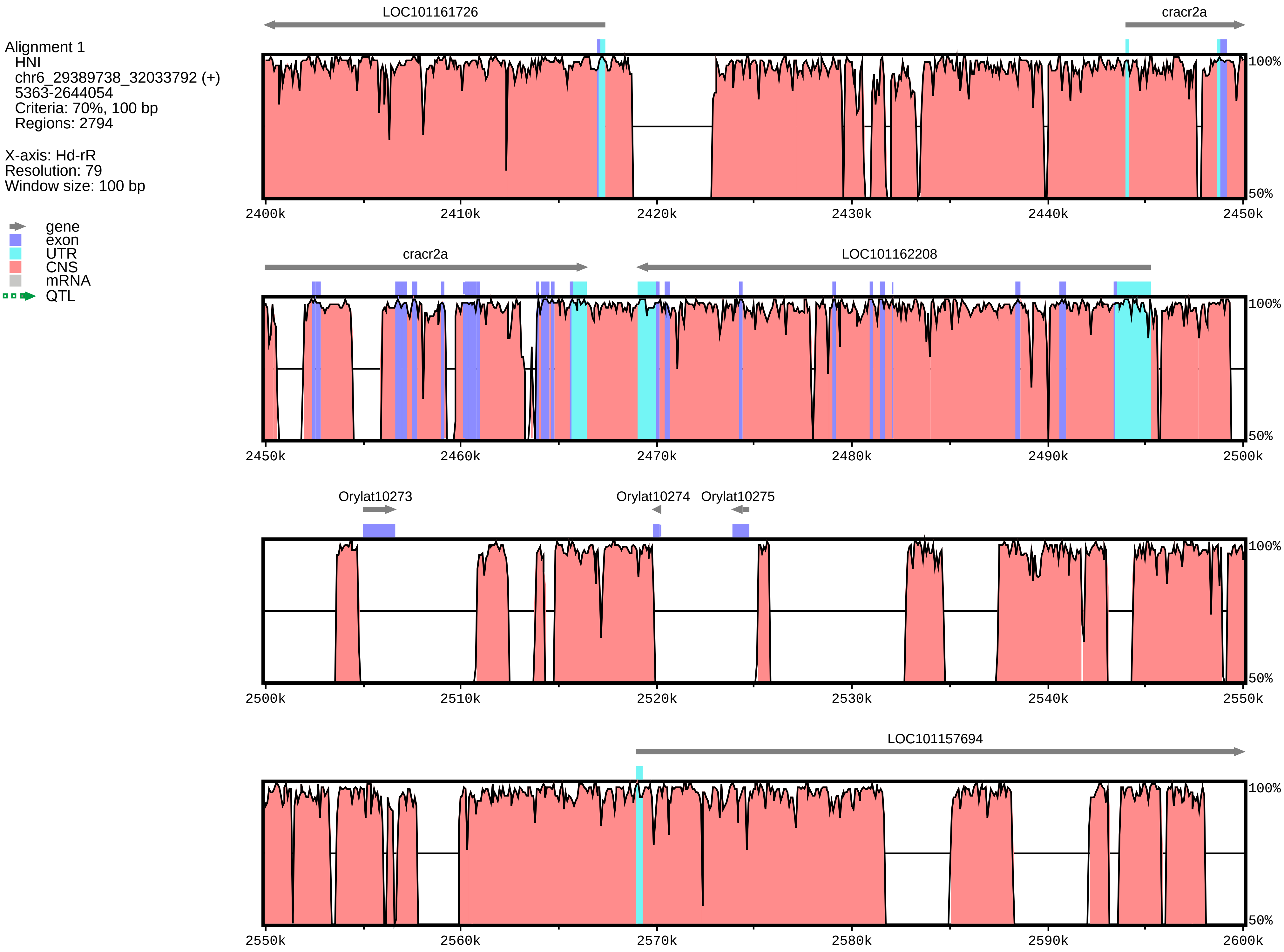

Alignment 1
HNI
chr6_29389738_32033792 (+)
5363-2644054
Criteria: 70%, 100 bp
Regions: 2794
X-axis: Hd-rR
Resolution: 79
Window size: 100 bp
gene
exon
UTR
CNS
mRNA
QTL
LOC101161726
cracr2a
LOC101162208
Orylat10273
Orylat10274
Orylat10275
LOC101157694
100%
50%
2400k
2410k
2420k
2430k
2440k
2450k
2460k
2470k
2480k
2490k
2500k
2510k
2520k
2530k
2540k
2550k
2560k
2570k
2580k
2590k
2600k

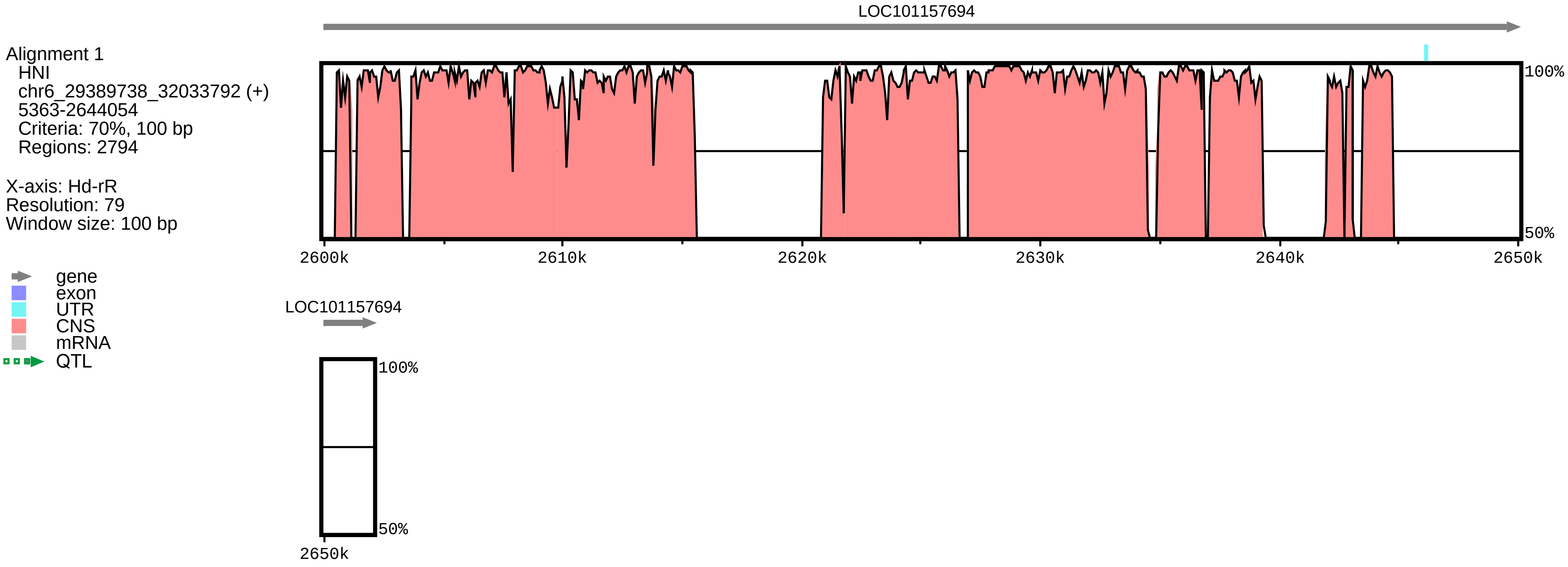

Supplementary Fig. 3

VISTA plot of the genomic region ±500 kbp for QTL6-1 to QTL6-3 (the HNI-II genome aligned to the HdrR-II1). The level of conservation (vertical axis) is shown in the coordinates of the HdrR-II1 sequence (horizontal axis). Conserved regions above the level of 70% per 100 bp are highlighted under the curve, with red indicating a conserved non-coding region, violet, a conserved exon, and blue, an untranslated region. Genes are represented by gray arrows, and QTLs represented by green arrows.

Supplementary Fig. 4

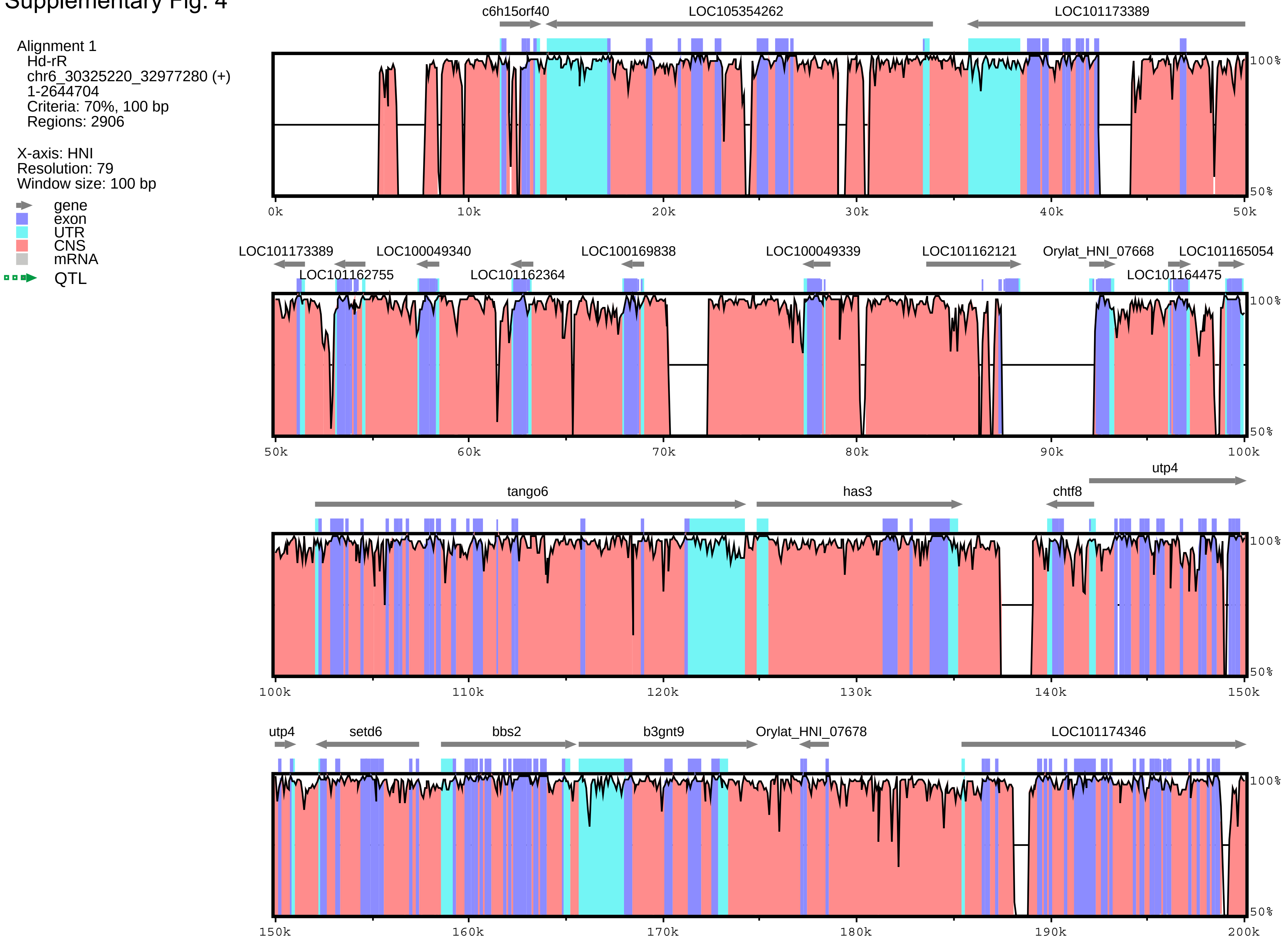

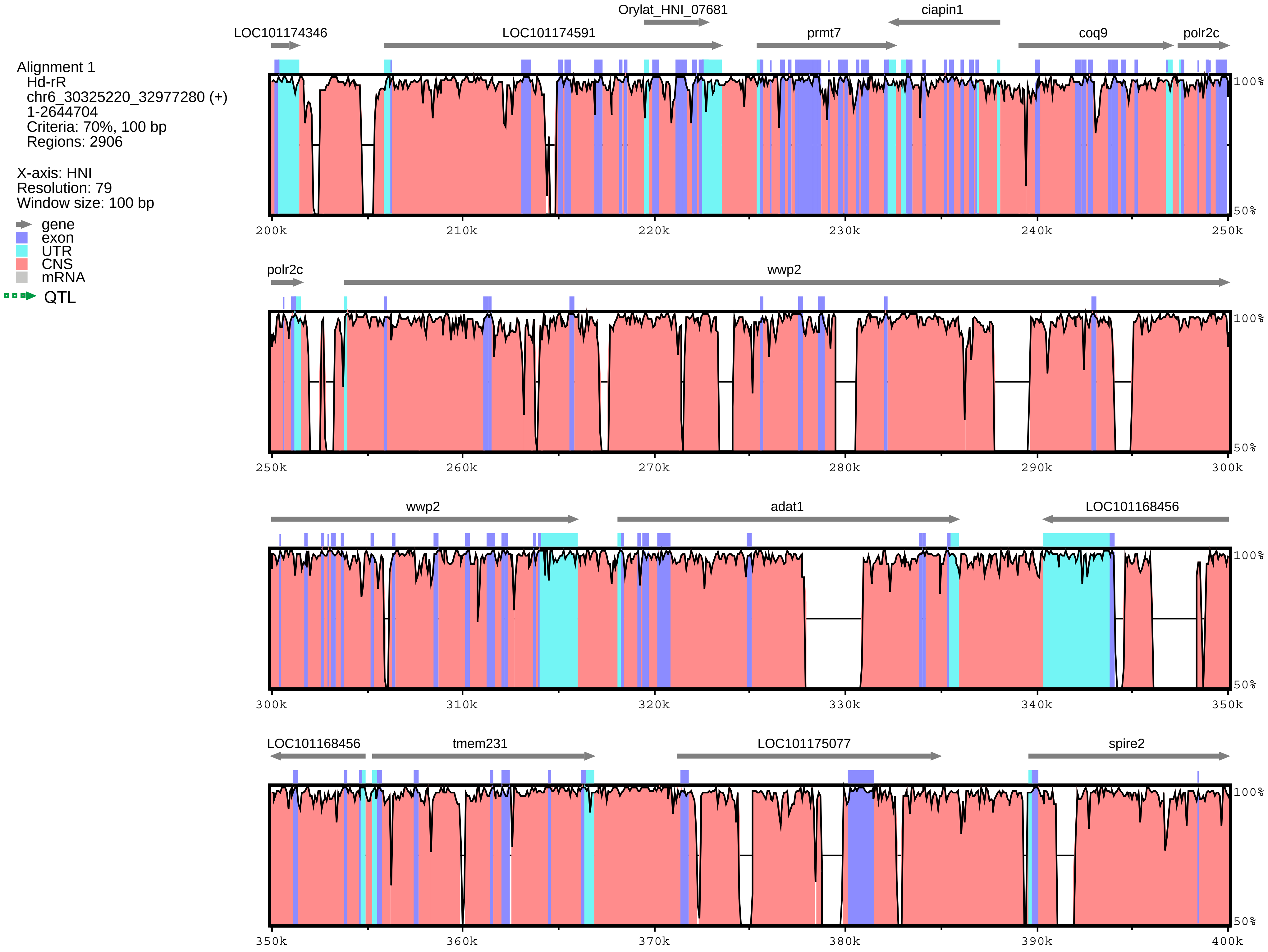
Alignment 1
Hd-rR
chr6_30325220_32977280 (+)
1-2644704
Criteria: 70%, 100 bp
Regions: 2906
X-axis: HNI
Resolution: 79
Window size: 100 bp
gene
exon
UTR
CNS
mRNA
QTL
Orylat_HNI_07681
ciapin1
LOC101174346
LOC101174591
prmt7
coq9
polr2c
100%
50%
200k
210k
220k
230k
240k
250k
polr2c
wwp2
260k
270k
280k
290k
300k
wwp2
adat1
LOC101168456
310k
320k
330k
340k
350k
LOC101168456
tmem231
LOC101175077
spire2
360k
370k
380k
390k
400k

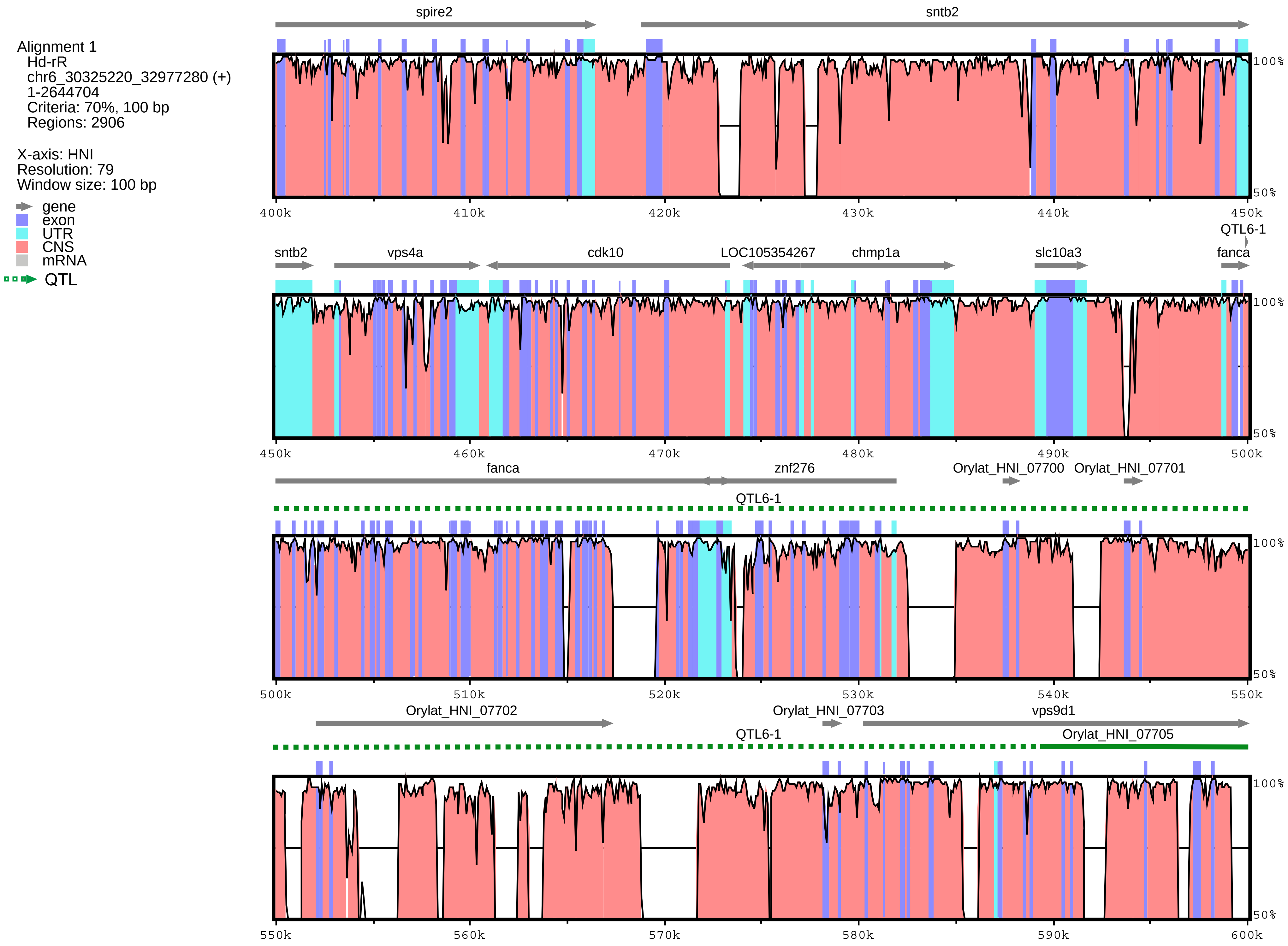

Alignment 1
Hd-rR
chr6_30325220_32977280 (+)
1-2644704
Criteria: 70%, 100 bp
Regions: 2906
X-axis: HNI
Resolution: 79
Window size: 100 bp
gene
exon
UTR
CNS
mRNA
QTL
spire2
sntb2
100%
50%
400k
410k
420k
430k
440k
450k
QTL6-1
sntb2
vps4a
cdk10
LOC105354267
chmp1a
slc10a3
fanca
460k
470k
480k
490k
500k
fanca
znf276
Orylat_HNI_07700
Orylat_HNI_07701
QTL6-1
510k
520k
530k
540k
550k
Orylat_HNI_07702
Orylat_HNI_07703
vps9d1
QTL6-1
Orylat_HNI_07705
560k
570k
580k
590k
600k

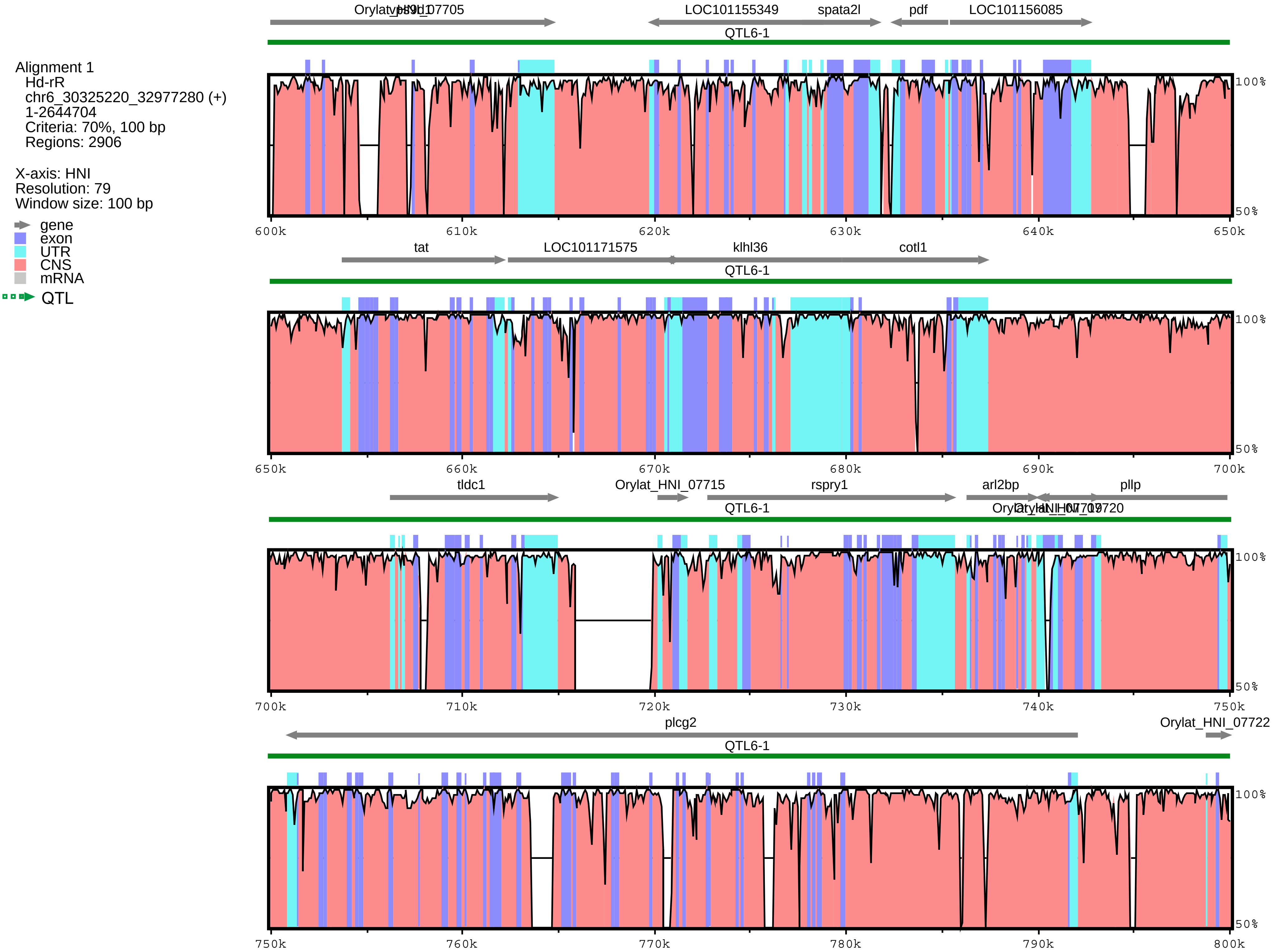

Alignment 1
Hd-rR
chr6_30325220_32977280 (+)
1-2644704
Criteria: 70%, 100 bp
Regions: 2906
X-axis: HNI
Resolution: 79
Window size: 100 bp
gene
exon
UTR
CNS
mRNA
QTL
LOC101155349
spata2l
pdf
LOC101156085
QTL6-1
100%
50%
600k
610k
620k
630k
640k
650k
tat
LOC101171575
klhl36
cotl1
660k
670k
680k
690k
700k
tldc1
Orylat_HNI_07715
rspry1
arl2bp
pllp
710k
720k
730k
740k
750k
plcg2
Orylat_HNI_07722
760k
770k
780k
790k
800k

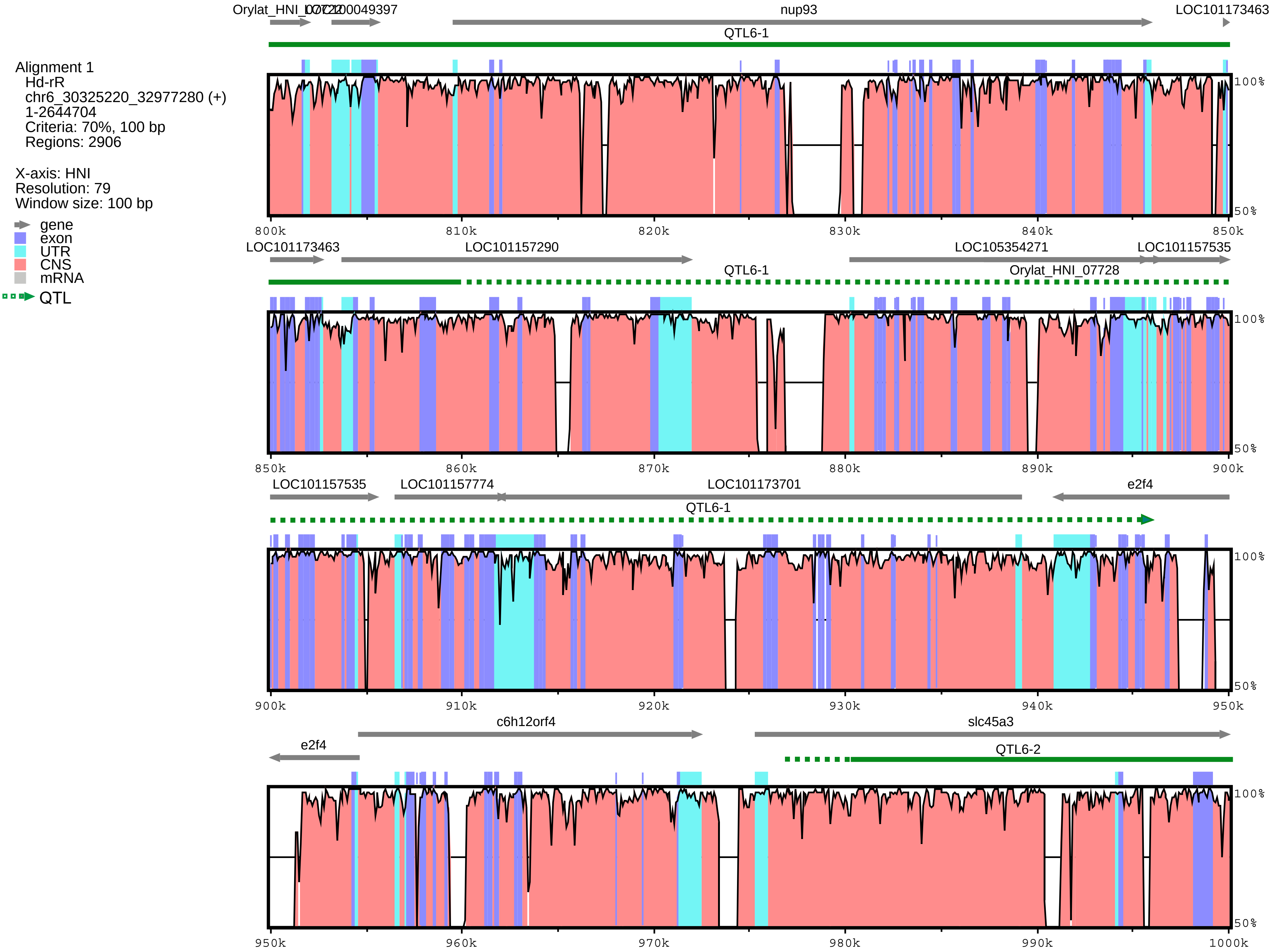

Alignment 1
Hd-rR
chr6_30325220_32977280 (+)
1-2644704
Criteria: 70%, 100 bp
Regions: 2906
X-axis: HNI
Resolution: 79
Window size: 100 bp
gene
exon
UTR
CNS
mRNA
QTL
nup93
LOC101173463
QTL6-1
LOC101157290
LOC105354271
LOC101157535
Orylat_HNI_07728
LOC101157774
LOC101173701
e2f4
c6h12orf4
slc45a3
QTL6-2
100%
50%
800k
810k
820k
830k
840k
850k
860k
870k
880k
890k
900k
910k
920k
930k
940k
950k
960k
970k
980k
990k
1000k

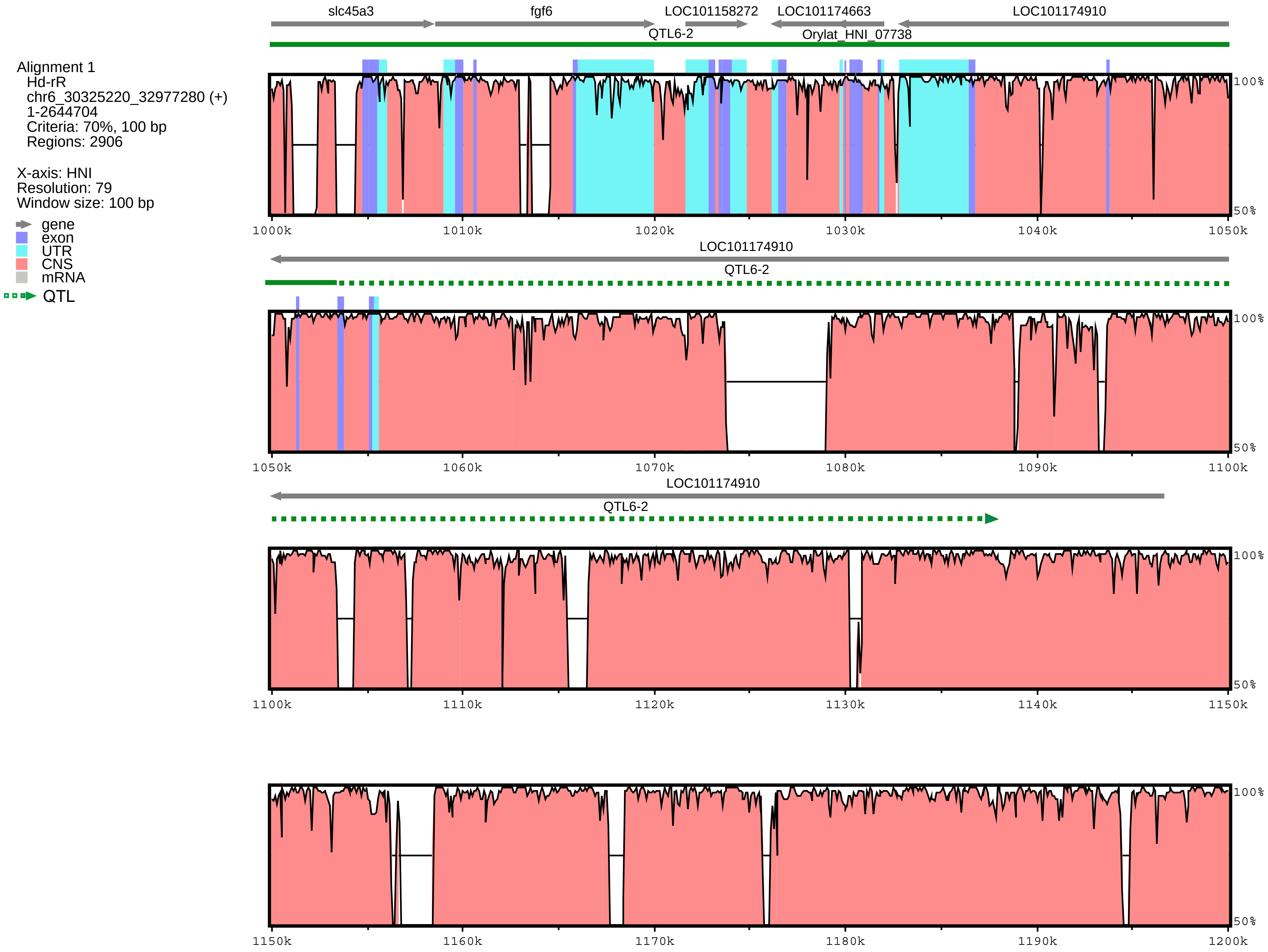

slc45a3
fgf6
LOC101158272
LOC101174663
LOC101174910
QTL6-2
Orylat_HNI_07738
Alignment 1
Hd-rR
chr6_30325220_32977280 (+)
1-2644704
Criteria: 70%, 100 bp
Regions: 2906
X-axis: HNI
Resolution: 79
Window size: 100 bp
gene
exon
UTR
CNS
mRNA
QTL
100%
50%
1000k
1010k
1020k
1030k
1040k
1050k
LOC101174910
QTL6-2
1060k
1070k
1080k
1090k
1100k
LOC101174910
QTL6-2
1110k
1120k
1130k
1140k
1150k
1160k
1170k
1180k
1190k
1200k

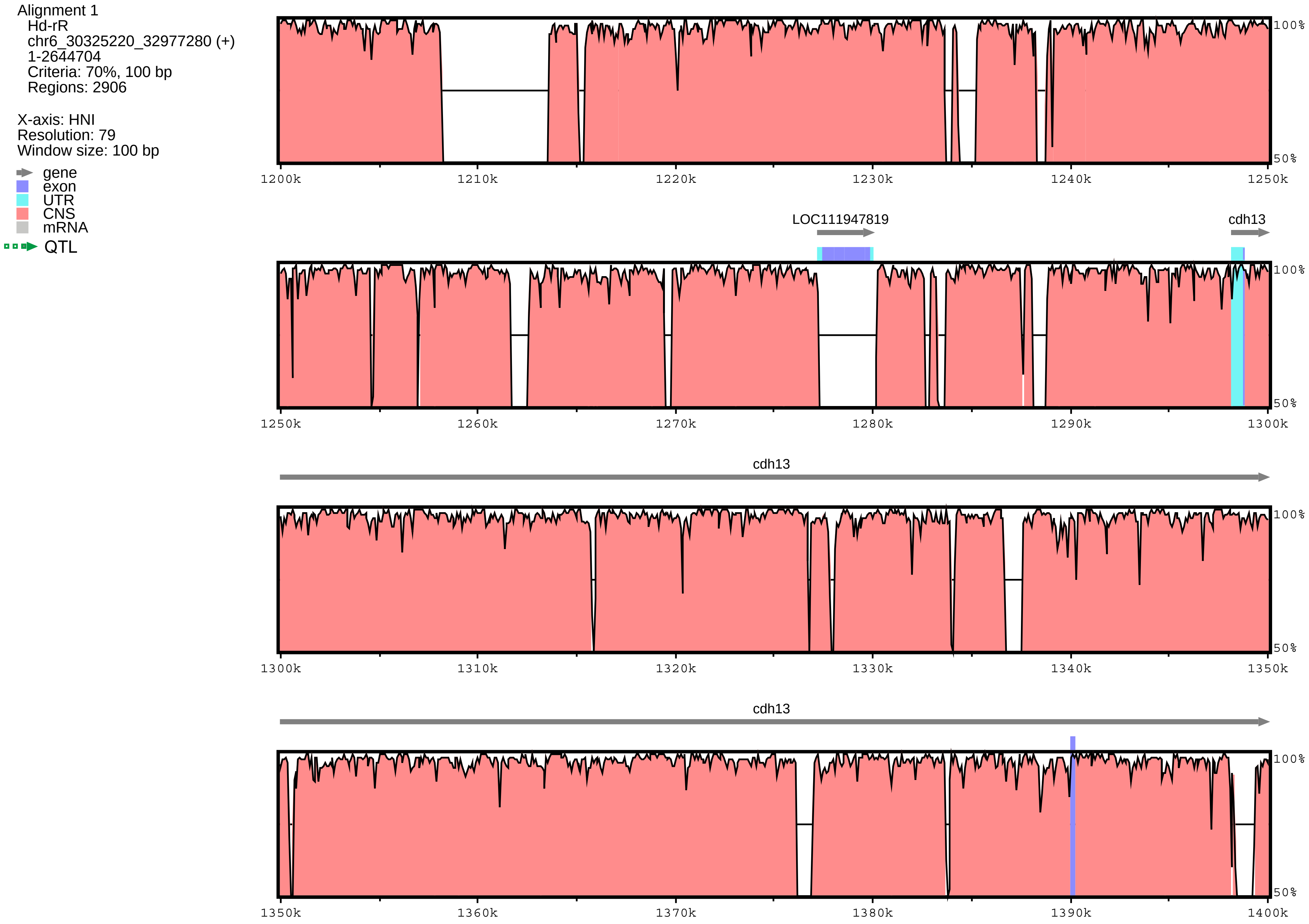

Alignment 1
Hd-rR
chr6_30325220_32977280 (+)
1-2644704
Criteria: 70%, 100 bp
Regions: 2906
X-axis: HNI
Resolution: 79
Window size: 100 bp
gene
exon
UTR
CNS
mRNA
QTL
100%
50%
1200k
1210k
1220k
1230k
1240k
1250k
LOC111947819
cdh13
1260k
1270k
1280k
1290k
1300k
1310k
1320k
1330k
1340k
1350k
1360k
1370k
1380k
1390k
1400k

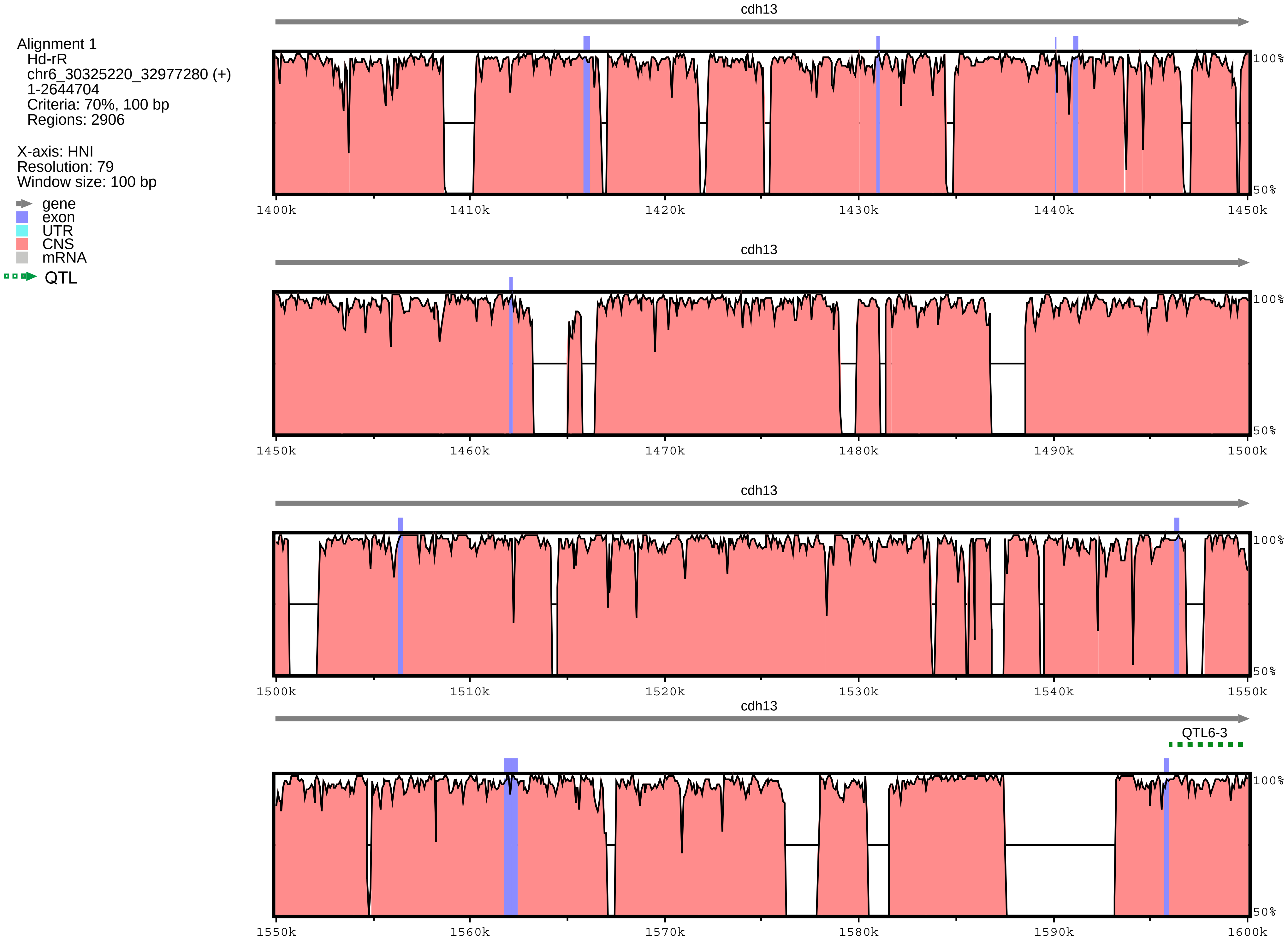

Alignment 1
Hd-rR
chr6_30325220_32977280 (+)
1-2644704
Criteria: 70%, 100 bp
Regions: 2906
X-axis: HNI
Resolution: 79
Window size: 100 bp
gene
exon
UTR
CNS
mRNA
QTL
cdh13
100%
50%
1400k
1410k
1420k
1430k
1440k
1450k
cdh13
1450k
1460k
1470k
1480k
1490k
1500k
cdh13
1500k
1510k
1520k
1530k
1540k
1550k
cdh13
QTL6-3
1550k
1560k
1570k
1580k
1590k
1600k

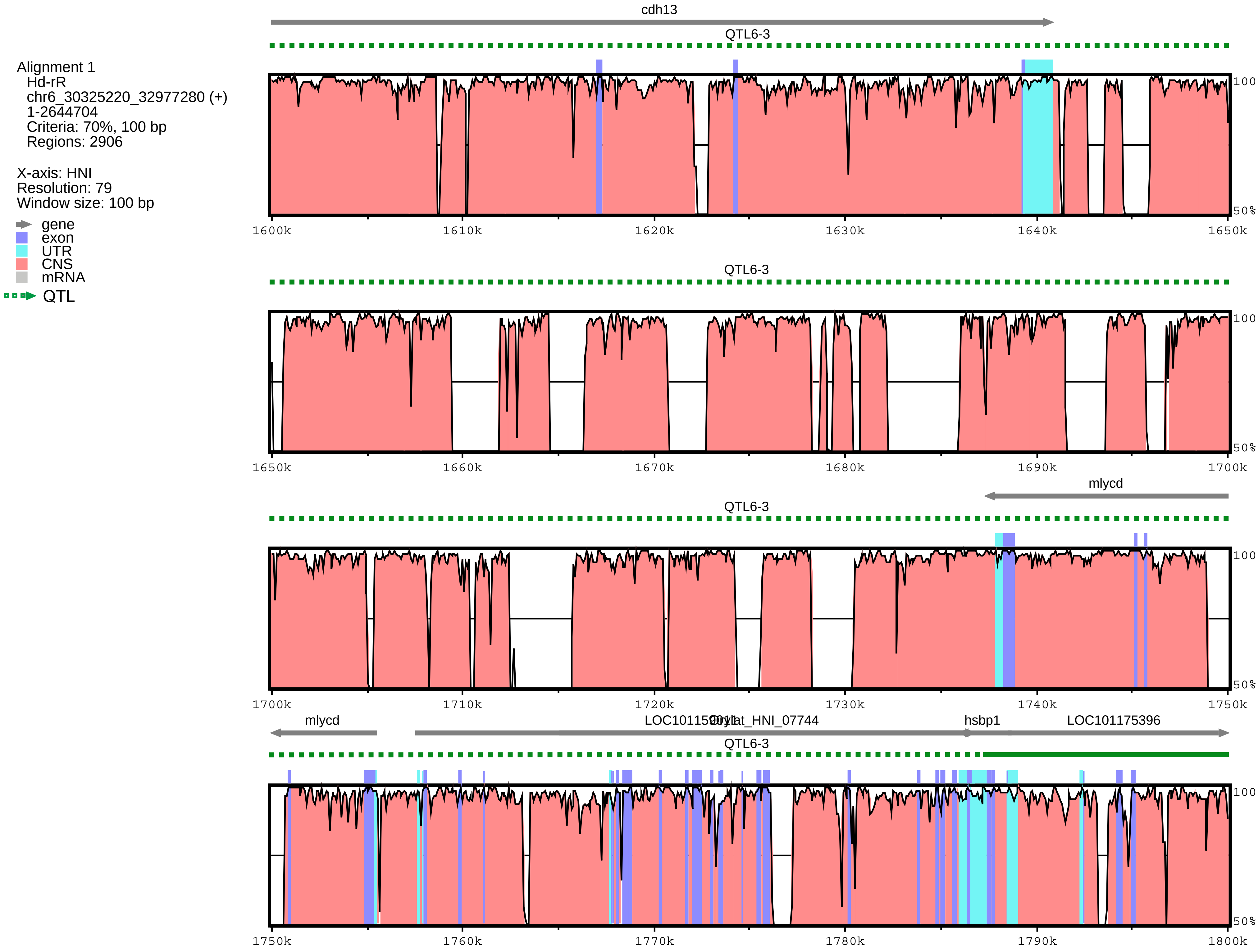

Alignment 1
Hd-rR
chr6_30325220_32977280 (+)
1-2644704
Criteria: 70%, 100 bp
Regions: 2906
X-axis: HNI
Resolution: 79
Window size: 100 bp
gene
exon
UTR
CNS
mRNA
QTL
cdh13
QTL6-3
100%
50%
1600k
1610k
1620k
1630k
1640k
1650k
1660k
1670k
1680k
1690k
1700k
mlycd
1710k
1720k
1730k
1740k
1750k
LOC101159011
Orylat_HNI_07744
hsbp1
LOC101175396
1760k
1770k
1780k
1790k
1800k

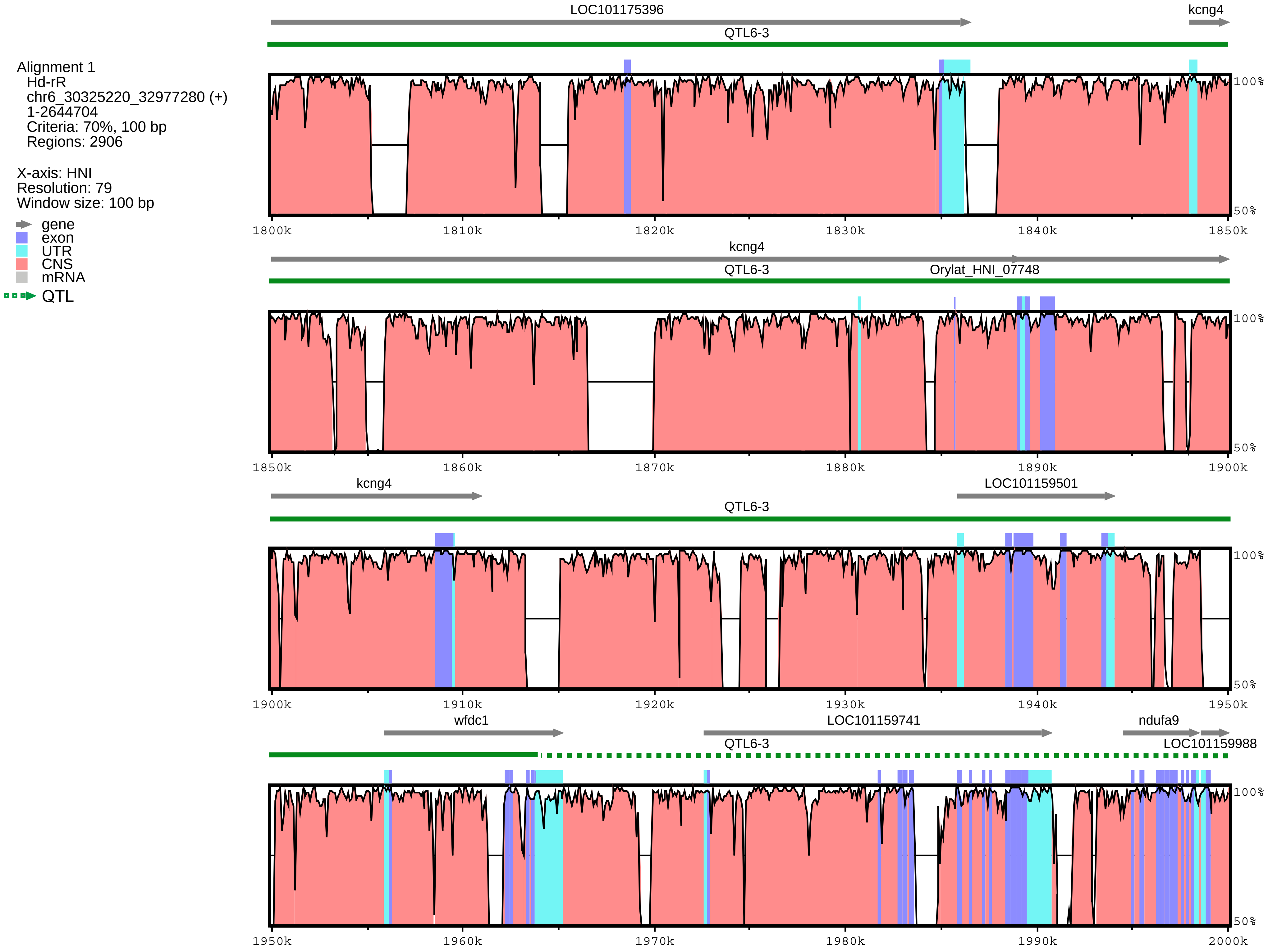

Alignment 1
Hd-rR
chr6_30325220_32977280 (+)
1-2644704
Criteria: 70%, 100 bp
Regions: 2906
X-axis: HNI
Resolution: 79
Window size: 100 bp
gene
exon
UTR
CNS
mRNA
QTL
LOC101175396
kcng4
QTL6-3
100%
50%
1800k
1810k
1820k
1830k
1840k
1850k
kcng4
QTL6-3
Orylat_HNI_07748
1850k
1860k
1870k
1880k
1890k
1900k
kcng4
LOC101159501
QTL6-3
1900k
1910k
1920k
1930k
1940k
1950k
wfdc1
LOC101159741
ndufa9
QTL6-3
LOC101159988
1950k
1960k
1970k
1980k
1990k
2000k

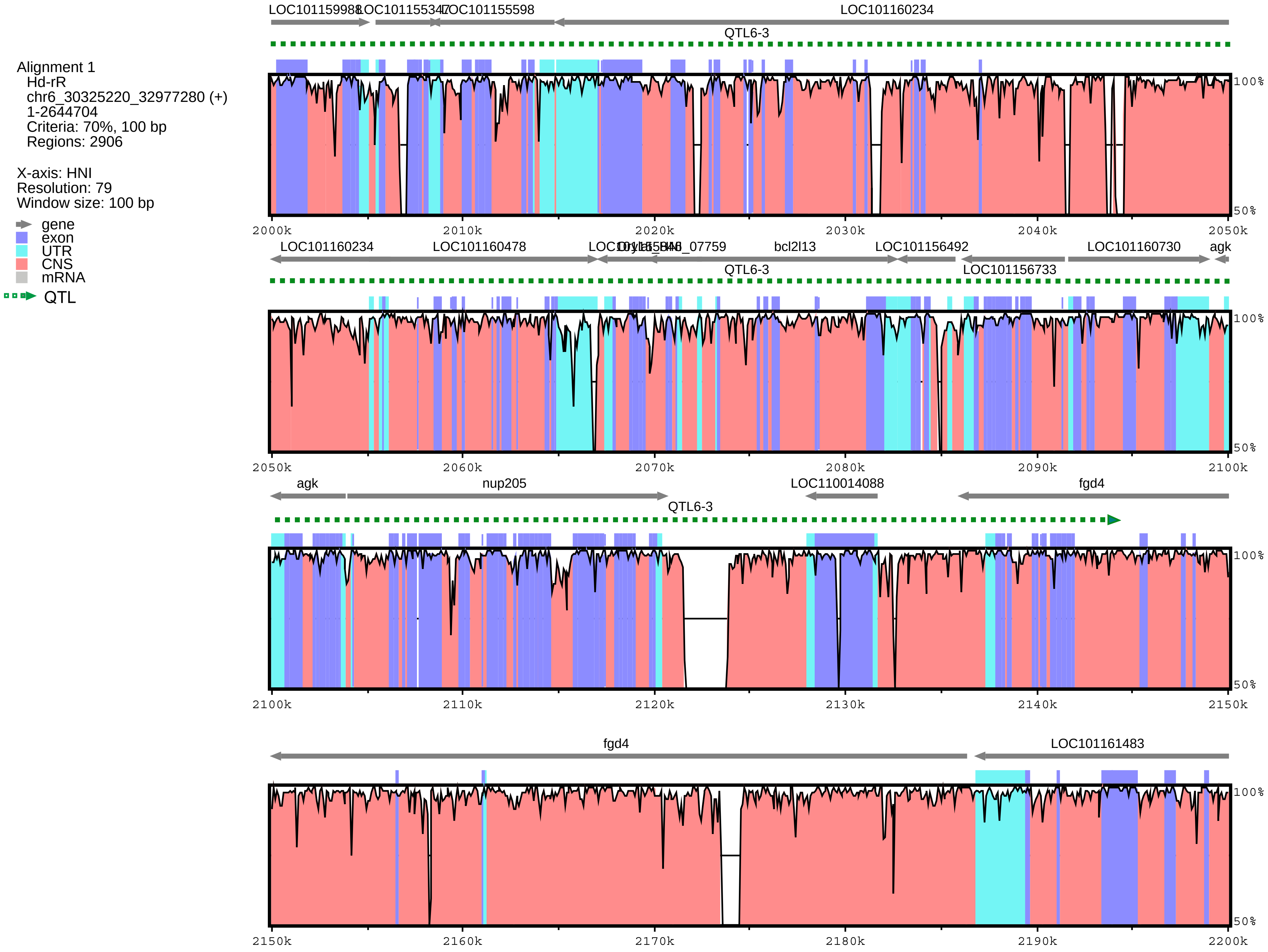

Alignment 1
Hd-rR
chr6_30325220_32977280 (+)
1-2644704
Criteria: 70%, 100 bp
Regions: 2906
X-axis: HNI
Resolution: 79
Window size: 100 bp
gene
exon
UTR
CNS
mRNA
QTL
LOC101159988
LOC101155598
LOC101160234
QTL6-3
100%
50%
2000k
2010k
2020k
2030k
2040k
2050k
LOC101160234
LOC101160478
bcl2l13
LOC101156492
LOC101160730
agk
LOC101156733
QTL6-3
2050k
2060k
2070k
2080k
2090k
2100k
agk
nup205
LOC110014088
fgd4
QTL6-3
2100k
2110k
2120k
2130k
2140k
2150k
fgd4
LOC101161483
2150k
2160k
2170k
2180k
2190k
2200k

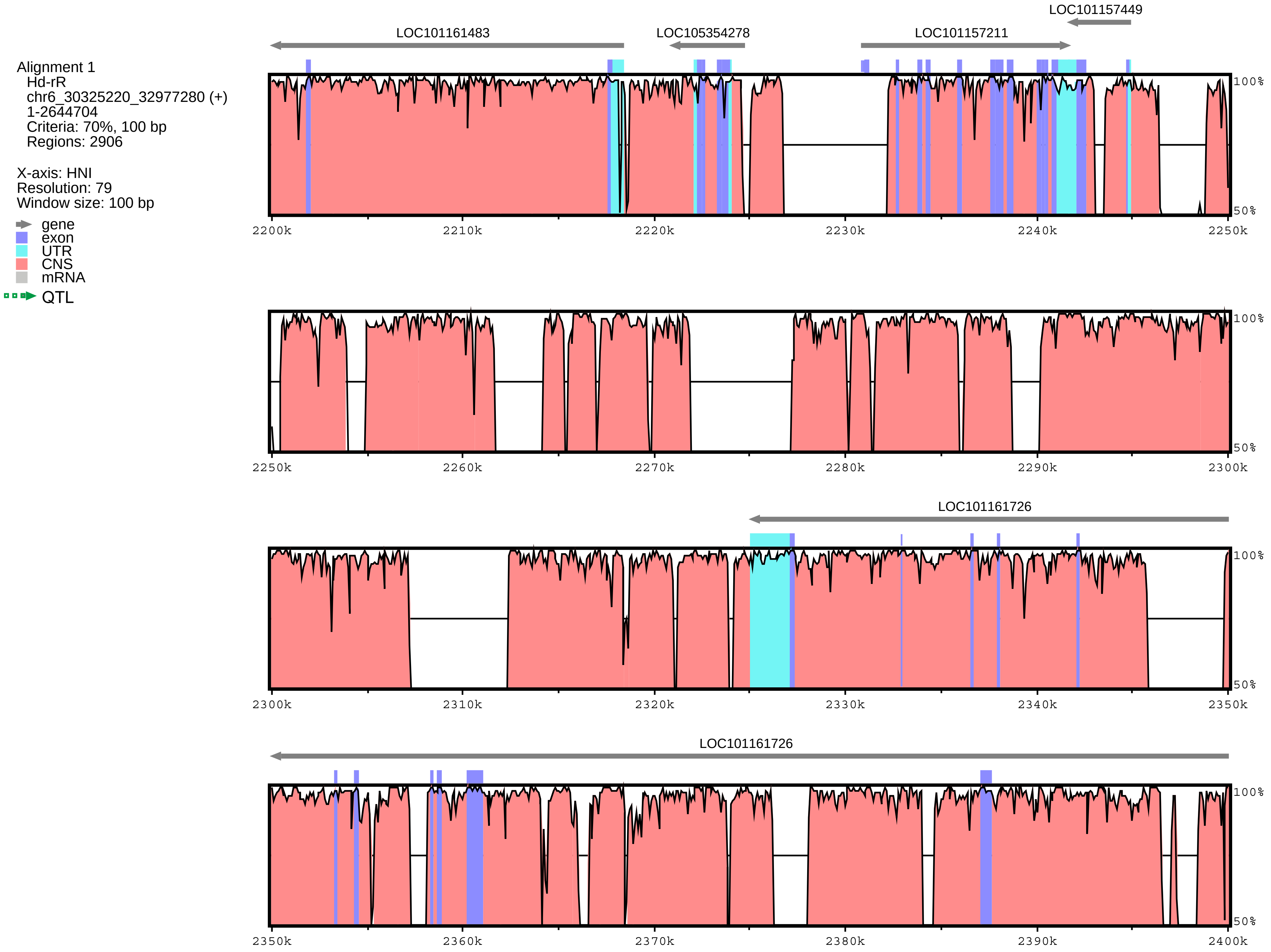

Alignment 1
Hd-rR
chr6_30325220_32977280 (+)
1-2644704
Criteria: 70%, 100 bp
Regions: 2906
X-axis: HNI
Resolution: 79
Window size: 100 bp
gene
exon
UTR
CNS
mRNA
QTL
LOC101161483
LOC105354278
LOC101157211
LOC101157449
LOC101161726
LOC101161726
100%
50%
2200k
2210k
2220k
2230k
2240k
2250k
2260k
2270k
2280k
2290k
2300k
2310k
2320k
2330k
2340k
2350k
2360k
2370k
2380k
2390k
2400k

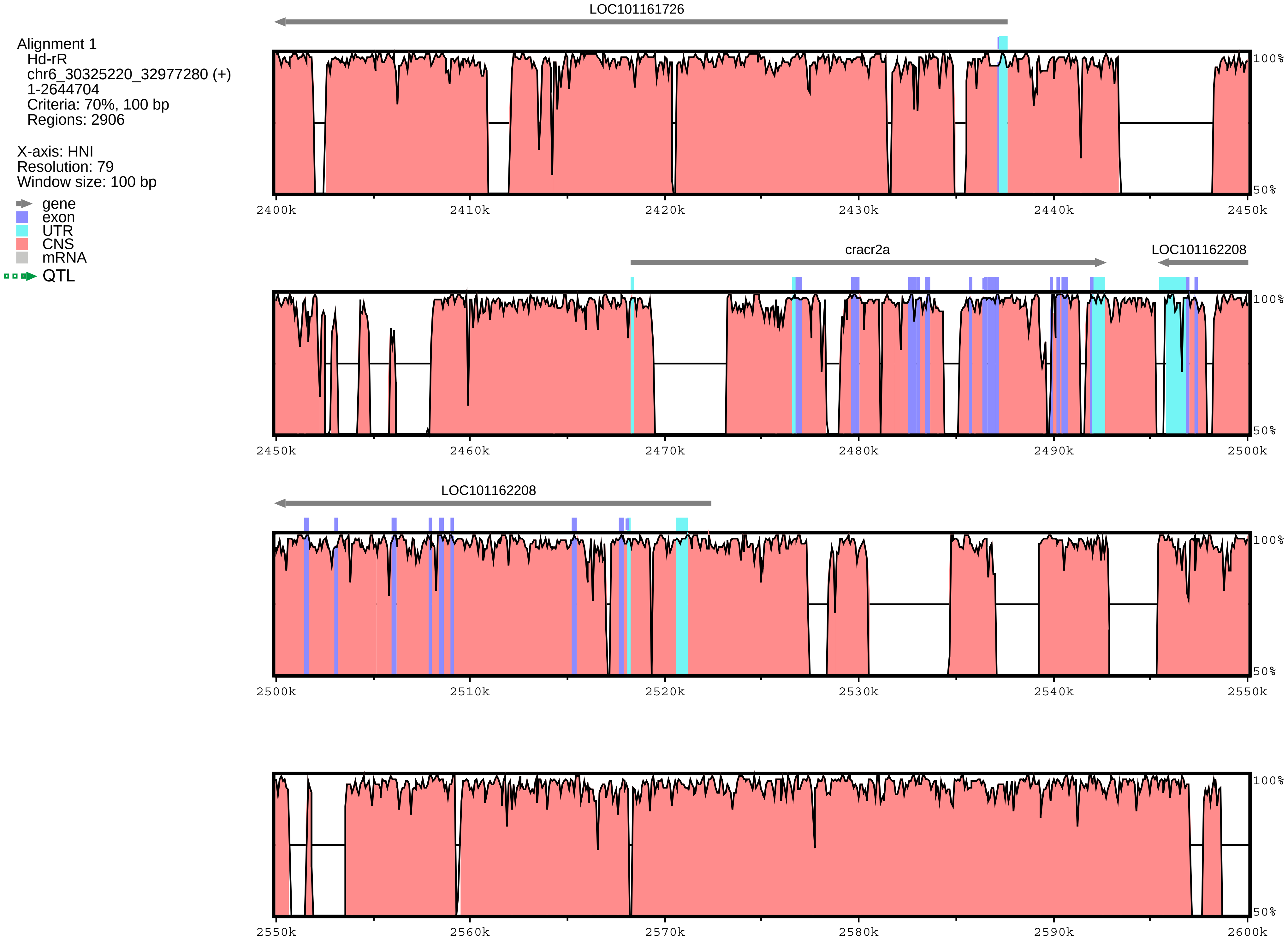

Alignment 1
Hd-rR
chr6_30325220_32977280 (+)
1-2644704
Criteria: 70%, 100 bp
Regions: 2906
X-axis: HNI
Resolution: 79
Window size: 100 bp
gene
exon
UTR
CNS
mRNA
QTL
LOC101161726
cracr2a
LOC101162208
LOC101162208
100%
50%
2400k
2410k
2420k
2430k
2440k
2450k
2460k
2470k
2480k
2490k
2500k
2510k
2520k
2530k
2540k
2550k
2560k
2570k
2580k
2590k
2600k

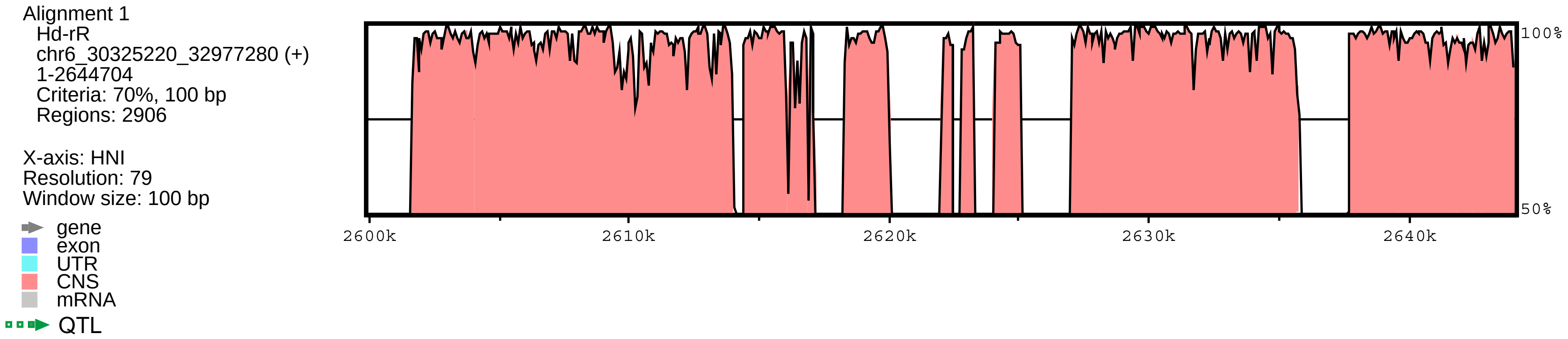

Supplementary Fig. 4

VISTA plot of the genomic region ±500 kbp for QTL6-1 to 6-3 (the HdrR-II1 genome aligned to the HNI-II). The level of conservation (vertical axis) is shown in the coordinates of the HNI-II sequence (horizontal axis). Conserved regions above the level of 70% per 100 bp are highlighted under the curve, with red indicating a conserved non-coding region, violet, a conserved exon, and blue, an untranslated region. Genes are represented by gray arrows, and QTLs represented by green arrows.
